# Lower Severity, Denser Network: Profile-Specific Symptom Architecture in Adolescent Internalizing and Externalizing Problems

**DOI:** 10.3390/bs16091643

**Published:** 2026-09-14

**Authors:** Huina Teng, Cui Zhou, Yue Li, Boyu Qiu, Wei Zhang

**Affiliations:** 1 Philosophy and Social Science Laboratory of Reading and Development in Children and Adolescents, South China Normal University, Ministry of Education, Guangzhou 510631, China; 2 School of Health Management, Guangzhou Medical University, Guangzhou 511436, China; 3 School of Psychology, South China Normal University, Guangzhou 510631, China; 4 Department of Psychology, Sun Yat-sen University, Guangzhou 510006, China

**Keywords:** internalizing problems, externalizing problems, latent profile analysis, network analysis, adolescents

## Abstract

**Introduction:** This study examined whether severity-defined profiles of internalizing and externalizing problems among Chinese adolescents differed in their symptom connectivity. **Method:** Participants were 1200 Chinese adolescents aged 9 to 15 years who were assessed in 2018 and 2022. Fourteen internalizing and externalizing problem items were analyzed. Latent profile analysis was used to obtain a coarse severity-based grouping at each wave, after which Gaussian graphical models were estimated within profiles. Profile networks were formally compared using permutation-based Network Comparison Tests, and robustness to the EBIC hyperparameter was examined across γ = 0, 0.25, and 0.50. **Results:** A two-profile solution was retained at each wave as an interpretable lower- versus higher-symptom partition with adequate group sizes. Formal comparisons showed greater global strength in the lower-symptom profile at both waves. Overall network structure differed significantly between profiles at Time 1 but not at Time 2. Several node strength differences were detected, although the exact ordering of central nodes was not uniformly robust across regularization settings. **Conclusions:** Severity-defined profiles differed reliably in overall symptom connectivity, whereas evidence for broader profile-specific network configurations was wave-specific. These cross-sectional network findings describe conditional association patterns and should be treated as hypothesis-generating rather than as evidence of causal mechanisms or optimal intervention targets.

## 1. Lower Severity, Denser Network: Profile-Specific Symptom Architecture in Adolescent Internalizing and Externalizing Problems

A central debate in psychopathology concerns whether comorbidity between internalizing and externalizing problems reflects a single underlying liability or whether these domains retain meaningful specificity. Caspi and Moffitt (2018) argued that a general psychopathology factor, which they termed the p-factor, captures the shared variance across all mental disorders, positioning severity as the primary organizing dimension of psychopathology. This unifying framework has gained substantial empirical support, with hierarchical models demonstrating that a higher-order factor accounts for comorbidity among internalizing and externalizing conditions in both adult and child samples (Krueger & Eaton, 2015; Martel et al., 2017). However, critics have questioned whether the p-factor represents a substantive latent construct or merely a statistical artifact. Fried et al. (2021) contended that the p-factor is currently “the sum of its parts,” reflecting shared method variance rather than a meaningful entity. Moreover, Brislin et al. (2022) showed that specific internalizing and externalizing factors exhibit differentiated nomological networks even after accounting for the p-factor, suggesting that severity alone cannot fully capture the organization of psychopathology. These competing perspectives imply that, while severity matters, qualitative differences in symptom organization may exist across severity levels, a question that symptom-level network approaches are uniquely positioned to address.

Network theory reconceptualizes psychopathology as emerging from interacting symptoms rather than from underlying latent diseases (Borsboom, 2017). In this framework, symptoms are represented as nodes in a network. Edges capture the conditional associations between symptoms, taking into account all the other symptoms in the network (Ryan et al., 2022). Certain symptoms, referred to as central symptoms, occupy highly connected positions and may activate and sustain other symptoms in the network (Fried et al., 2017). Centrality indices, particularly strength centrality, quantify the importance of individual symptoms within the network and have been used to identify potential intervention targets (Bringmann et al., 2019). Network theory permits symptom relations to be heterogeneous but does not require network architecture to change with severity. Whether severity-defined groups differ in symptom connectivity is therefore an empirical question rather than a theoretical implication. However, a question remains unresolved: whether network architecture changes qualitatively with severity, or whether higher severity simply reflects “more of the same” symptom organization. Despite rapid growth in psychopathological network research, few studies have estimated symptom networks within empirically derived severity subgroups (Forbes et al., 2019). However, recent work has begun to examine differences in symptom networks across severity levels (Ebrahimi et al., 2024).

Latent profile analysis (LPA) is a person-centered statistical approach that identifies unobserved subgroups of individuals who share similar response patterns on continuous indicators. When applied to psychopathology data, LPA typically reveals two to three profiles distinguished primarily by overall symptom severity, with a lower-severity profile and a higher-severity profile emerging as the most common solution (Clark & Malecki, 2022; Eastman et al., 2018). LPA has been widely used to study the heterogeneity of adolescent behavioral and emotional problems, including comorbidity between internalizing and externalizing symptoms (Brinkman et al., 2016; Zhao et al., 2019). Critically, LPA provides the person-level heterogeneity that within-profile network analysis requires. By assigning individuals to empirically derived subgroups, researchers can estimate separate symptom networks for each profile and compare their architectures directly. Longitudinal data enrich this approach by revealing temporal dynamics, specifically the stability of profile membership over time and the probability of transitioning between profiles.

The present study had two aims. First, we used latent profile analysis to derive an interpretable grouping of adolescents based on internalizing and externalizing symptom levels at each wave and described transitions in profile membership over the four-year interval. Second, we estimated profile-specific Gaussian graphical models and formally tested whether the lower- and higher-symptom profiles differed in overall connectivity, network structure, individual edges, and node strength. Because neither the p-factor nor network theory entails a necessary prediction about network invariance across severity groups, these network comparisons were treated as empirical and partly exploratory. We additionally examined whether the results were robust to alternative EBIC regularization settings.

## 2. Methods

### 2.1. Participants

Data were drawn from the China Family Panel Studies (CFPS), a nationally representative longitudinal survey conducted by the Institute of Social Science Survey at Peking University. The CFPS uses a multistage probability proportional to size sampling strategy to select households across 25 provinces in China. The present study used two waves of data collected in 2018 (Time 1) and 2022 (Time 2). At Time 1, participants were 1200 adolescents aged 9 to 15 years (*M* = 12.12, *SD* = 1.55), of whom 646 (53.8%) were male and 554 (46.2%) were female. At Time 2, participants were aged 13 to 20 years (*M* = 16.11, *SD* = 1.55). The sex distribution remained consistent across waves (646 male, 554 female). One participant had a single missing item response at Time 2, which was imputed with the item mean, and no participants were excluded for missing data. For information on sample selection and attrition, see the Supplementary Materials.

### 2.2. Measures

Internalizing and externalizing problems were assessed using 14 items derived from the Child Behavior Checklist (Achenbach & Edelbrock, 1981) as implemented in the CFPS (Xie & Lu, 2015). The measure comprises two subscales: internalizing problems (eight items, referred to here as I1–I8) and externalizing problems (six items, referred to as E1–E6). Internalizing items assess symptoms of anxiety, depression, and withdrawal; an example item is “I often feel sad and upset”. Meanwhile, externalizing items capture aggressive behavior, rule-breaking, and attention problems; an example item is “I get in trouble for fighting with my peers”. Items were rated on a 5-point scale (1 = *completely inconsistent*, 5 = *completely compliant*), with higher scores indicating greater severity. The correspondence between abbreviations and specific item contents is presented in Table 1. Previous research has supported the validity and reliability of the Child Behavior Checklist in Chinese adolescent samples (Cui et al., 2021). The alpha coefficients for the full 14-item measure in the current study were 0.79 (Time 1) and 0.81 (Time 2).

### 2.3. Data Analysis

All analyses were conducted in Python 3.12. Descriptive statistics, including means, standard deviations, and 95% confidence intervals, were computed for all 14 items at both time points. Normality was assessed using the Shapiro–Wilk test. Bivariate associations among items were examined using Pearson or Spearman correlations depending on distributional properties.

LPA was conducted using StepMix with an expectation-maximization algorithm. Models with one to five latent profiles were estimated, assuming Gaussian mixtures with diagonal covariance matrices. Each model was fitted with 10 random starts and a maximum of 500 iterations. Model fit was evaluated using the Akaike Information Criterion (AIC), Bayesian Information Criterion (BIC), sample-size-adjusted BIC (aBIC), and entropy. Solutions were additionally evaluated based on the minimum estimated item variance, class sizes, average posterior probabilities, and profile shapes, and solutions with variance estimates at the numerical lower bound were treated as singular. Sensitivity analyses included Gaussian models with item-specific variances constrained equal across profiles and latent class models treating the five-point indicators as categorical.

To examine longitudinal transitions in profile membership, a chi-square test of independence was performed. Cramér’s *V* was calculated as a measure of effect size. Row-standardized transition probabilities were computed to quantify the likelihood of transitioning between profiles across waves. Additionally, Kruskal–Wallis *H* tests were conducted to compare item scores across four transition groups (stable lower-severity profile, lower-to-higher, higher-to-lower, stable higher-severity profile) at both time points. Epsilon-squared was computed as a nonparametric effect size measure. Dunn’s post hoc tests were used for pairwise comparisons when omnibus tests were significant.

Within-profile network analysis was conducted using Gaussian graphical models (GGMs) estimated via the graphical LASSO with Extended Bayesian Information Criterion (EBIC; γ = 0.5) model selection (Epskamp et al., 2018a, 2018b). Networks were estimated separately for each profile at each wave, resulting in four networks. To assess tuning sensitivity, all four profile-specific networks were re-estimated using γ = 0 and γ = 0.25 in addition to γ = 0.50, and robustness was evaluated by comparing retained edge counts, global strength, edge-weight correlations, and strength rankings. Each network comprised 14 nodes representing the internalizing and externalizing items. Edges represent partial correlations between items after controlling for all other items. Centrality was assessed using strength, betweenness, and closeness indices. Network stability was evaluated using case-dropping bootstrap procedures with 1000 replications to compute correlation stability (CS) coefficients. CS values above 0.50 indicate stable centrality indices (Borsboom et al., 2021; Epskamp & Fried, 2018). Based on stability analyses, only strength centrality is reported in the main text. Betweenness and closeness, which were unstable for most profiles (CS < 0.50), are reported in the Supplementary Materials (Tables S3 and S4) but not in the manuscript.

### 2.4. Network Comparison Test

Lower- and higher-symptom profile networks were formally compared separately at each wave using the Network Comparison Test (NetworkComparisonTest version 2.2.3; Van Borkulo et al., 2023). Weighted Gaussian EBICglasso networks were estimated using γ = 0.50 and 5000 label permutations. Independent-sample permutations were used because the two profiles comprised disjoint groups within each wave. Omnibus tests evaluated network-structure invariance, defined by the maximum absolute difference between corresponding edges, and global-strength invariance, defined by the absolute difference in the sum of absolute edge weights. We also tested all 91 edges and the strength of all 14 nodes. Holm correction was applied separately within each wave to the edge-level and node-level tests. Rank differences not supported by these tests were interpreted descriptively.

## 3. Results

### 3.1. Descriptive Statistics

Descriptive statistics for all 14 items at Time 1 and Time 2 are presented in Table 2. At Time 1, mean scores ranged from 1.63 (E6, SD = 0.92) to 3.45 (I8, SD = 1.27). At Time 2, mean scores ranged from 1.45 (E6, SD = 0.79) to 3.49 (I8, SD = 1.24). All items exhibited significant departures from normality (Shapiro–Wilk’s *W* = 0.60–0.89, all *p* < 0.001). Bivariate correlations among items are reported in Tables S1 and S2 of the Supplementary Materials.

### 3.2. LPA

Fit indices for the one- to five-class solutions at both waves are presented in Table 3. At Time 1, the two-profile solution was retained as an interpretable lower- versus higher-symptom partition. The lower-severity profile comprised 552 adolescents (46.0%), and the higher-severity profile comprised 648 (54.0%). Descriptive statistical analysis is presented in Table 4. At Time 2, the two-profile solution was likewise retained, with 561 adolescents (46.8%) in the lower-severity profile and 639 (53.3%) in the higher-severity profile.

The three- through five-profile solutions contained item variances at the numerical lower bound, indicating singular solutions, so their likelihood gains and high entropy did not provide valid evidence for additional profiles. Information criteria also continued to improve in the equal-variance and categorical-indicator sensitivity models, and inspection of the alternative solutions showed that additional profiles generally subdivided the severity continuum or isolated small item-specific groups. The two-profile solution was therefore retained as a coarse lower- and higher-symptom partition with adequate sample sizes for network estimation. Detailed fit indices at Time 1 and Time 2 are presented in Table 3.

### 3.3. Profile Transitions

**Figure 1:**
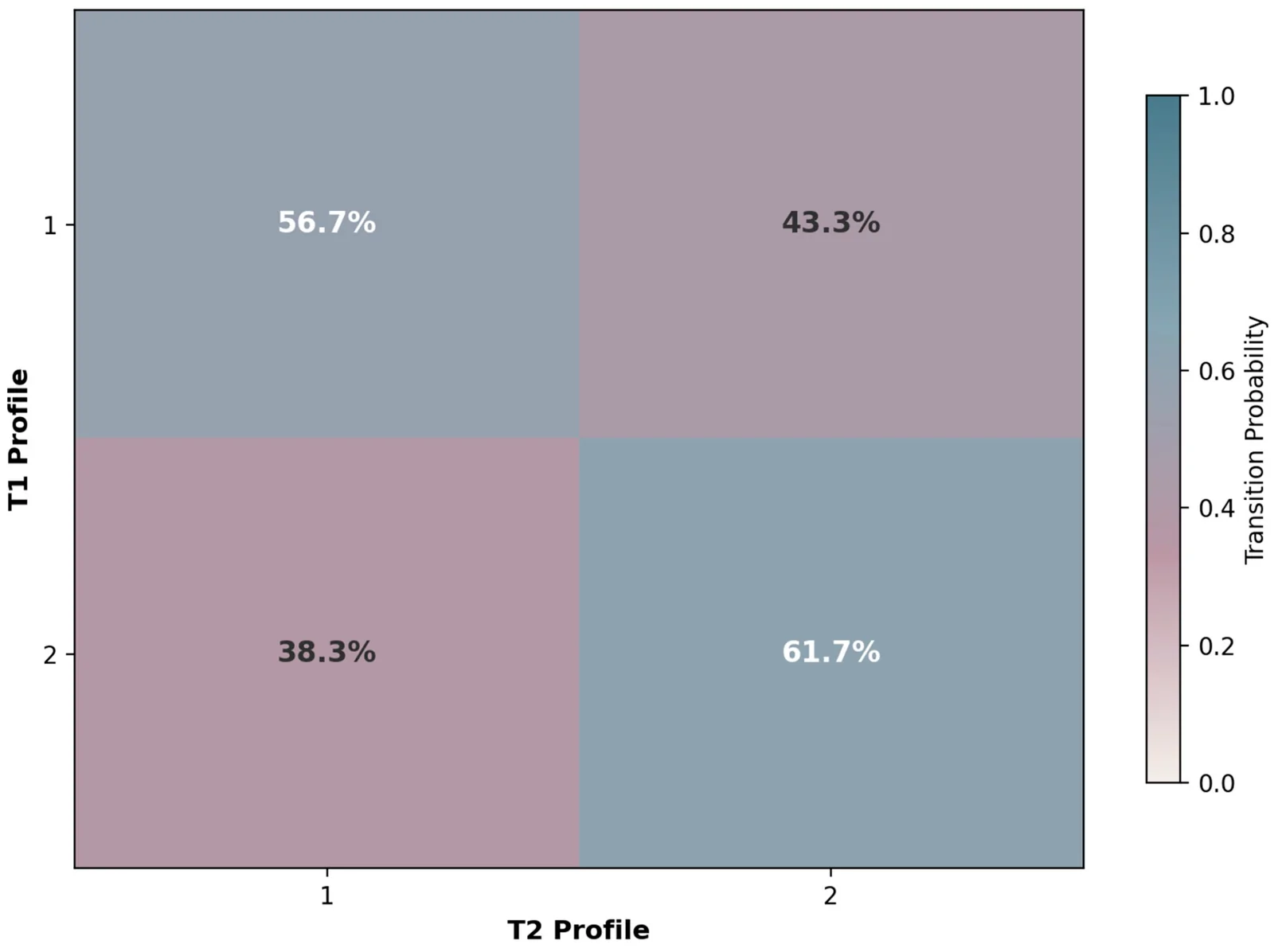
Profile transition heatmap. Note. Heatmap showing row-standardized transition probabilities from Time 1 to Time 2 profile membership. Color intensity represents the probability of transitioning between profiles. Values on the diagonal indicate stability (same profile at both waves). Lower-severity profile (Profile 1): 56.7% stable; higher-severity profile (Profile 2): 61.7% stable. Chi-square test: χ^2^(1) = 39.94, *p* < 0.001, Cramér’s *V* = 0.182.

Transition probabilities are presented in Table 5. The chi-square test revealed a significant association between profile membership at Time 1 and Time 2 (χ^2^(1) = 39.94, *p* < 0.001, Cramér’s *V* = 0.182). Among adolescents in the lower-severity profile at Time 1, 56.7% remained in that profile at Time 2, while 43.3% moved to the higher-severity profile. Among those in the higher-severity profile at Time 1, 61.7% remained, while 38.3% moved to the lower-severity profile. The transition heatmap is shown in Figure 1.

### 3.4. Difference Tests Across Transition Groups

Kruskal–Wallis *H* tests comparing item scores across four transition groups are presented in Table 6. At Time 1, all 14 items showed significant differences (all *p* < 0.001 except I8, *p* = 0.003), with effect sizes ranging from ε^2^ = 0.01 (I8) to 0.21 (I4, I5). At Time 2, all items were significant (all *p* < 0.001), with effect sizes from ε^2^ = 0.03 (I8) to 0.24 (I4). Post hoc analyses indicated that differences were primarily driven by between-profile contrasts (lower-origin vs. higher-origin), whereas within-origin groups (stable vs. transitioned) generally did not differ. Detailed post hoc results can be found in Table S3.

### 3.5. Within-Profile Network Analysis

**Figure 2:**
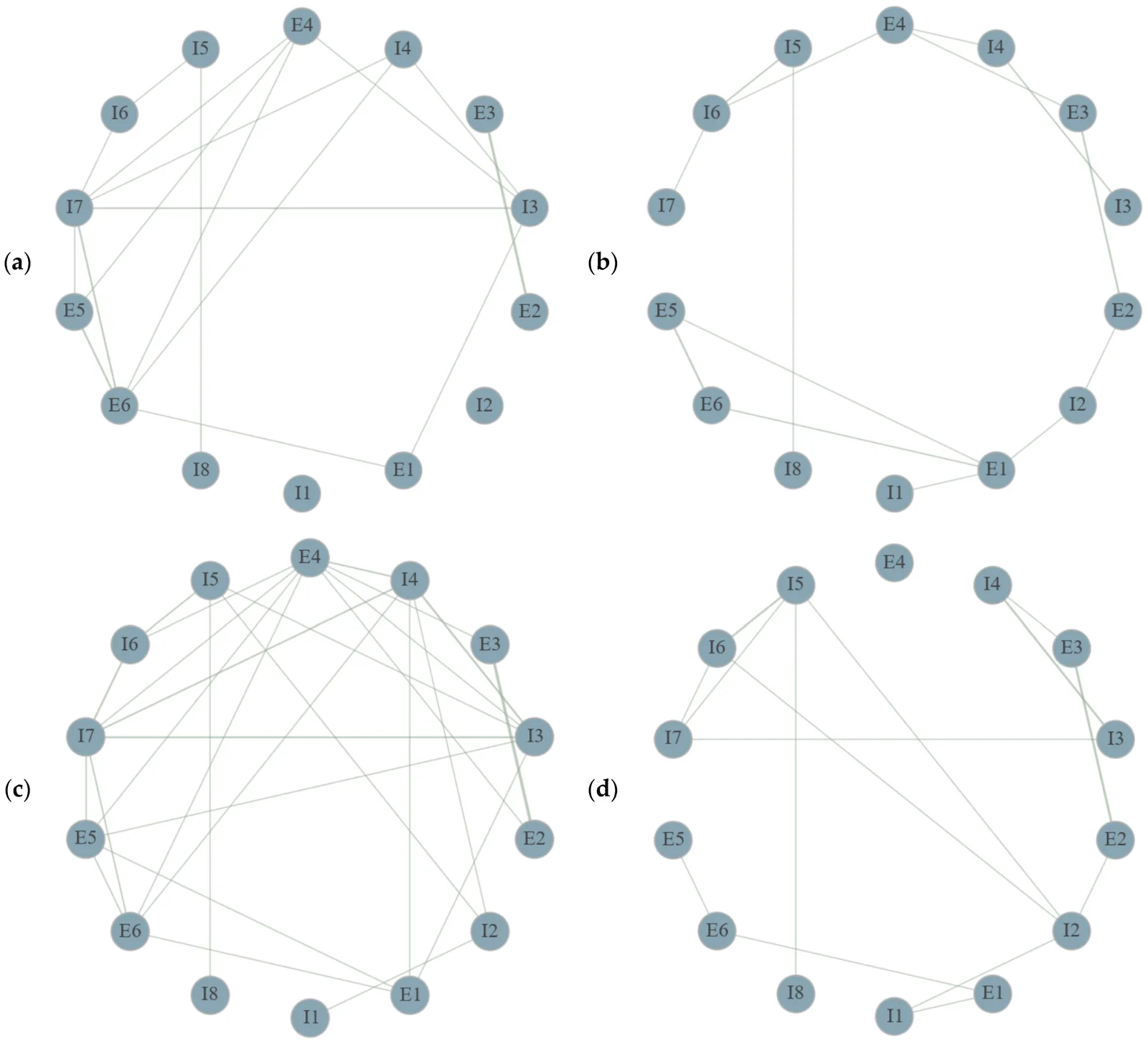
Within-profile network plots. Note. Gaussian graphical model networks for (**a**) lower-severity profile and (**b**) higher-severity profile at Time 1, and (**c**) lower-severity profile and (**d**) higher-severity profile at Time 2. Total of 14 nodes (eight internalizing [I1–I8], six externalizing [E1–E6]). Edges represent partial correlations; thicker and more saturated edges indicate stronger associations. Green edges indicate positive association.

Strength centrality values are presented in Table 7, and network plots are shown in Figure 2. In the lower-severity profile at Time 1 (17 edges), the most central items were I7 (strength = 0.86), E6 (0.77), and I3 (0.44). In the higher-severity profile at Time 1 (14 edges), the most central items were I6 (0.41), E6 (0.38), and E2 (0.34). At Time 2, the lower-severity profile network (28 edges) was dominated by I7 (1.05), I4 (0.93), and E4 (0.85), while the higher-severity profile network (15 edges) featured I5 (0.52), I2 (0.42), and I6 (0.42) as most central. CS coefficients for the centrality indices are presented in Figures S1 and S2 of the Supplementary Materials. The strength centrality was stable across all four profiles (CS = 0.77–0.94). Betweenness and closeness were unstable and are reported in the Supplementary Materials (Tables S4 and S5) but not in the manuscript.

The direction of the principal network-level pattern was robust across γ = 0, 0.25, and 0.50. The lower-symptom profile retained more edges than the higher-symptom profile at Time 1 (25 vs. 18, 23 vs. 14, and 17 vs. 14, respectively) and Time 2 (31 vs. 24, 28 vs. 18, and 28 vs. 15, respectively). Global strength was likewise consistently higher in the lower-symptom profile. Relative to γ = 0.50, edge-weight correlations ranged from 0.96 to 1.00, strength-rank correlations ranged from 0.93 to 1.00, and all commonly retained edges had the same sign. The leading nodes were broadly stable, although some second- and third-ranked nodes changed at γ = 0. Complete sensitivity results are reported in the Supplementary Materials.

### 3.6. Result of Network Comparison Test

Formal network comparisons yielded a qualified pattern. At Time 1, the omnibus network-structure test was significant (*M* = 0.219, *p* = 0.014). Global strength was also significantly higher in the lower-symptom profile than in the higher-symptom profile (2.596 vs. 0.850, *S* = 1.747, *p* = 0.004). At Time 2, the omnibus network-structure test was not significant (*M* = 0.183, *p* = 0.100), whereas global strength was again higher in the lower-symptom profile (4.258 vs. 1.846, *S* = 2.412, *p* < 0.001). Thus, overall connectivity differed consistently between profiles, but a broader difference in network configuration was supported only at Time 1.

After Holm correction across 91 edge tests, I7–E6 differed at Time 1 (lower = 0.219, higher = 0, adjusted *p* = 0.018). I4–E4 differed at Time 2 (lower = 0.183, higher = 0, adjusted *p* = 0.036), although this isolated result was treated as exploratory because the Time 2 omnibus structure test was not significant. Holm-corrected node tests showed greater strength in the lower-symptom profile for I7 and E6 at Time 1 (both adjusted *p* = 0.003) and for I3, I4, E4, I7, E5, and E6 at Time 2 (adjusted *p* = 0.003–0.007). All other edge and node differences were nonsignificant after correction. Complete Network Comparison Test results, including the omnibus tests, all Holm-corrected edge and node strength comparisons, and the permutation distributions, are provided in the Supplementary Materials.

## 4. Discussion

The lower-symptom profile had greater global strength at both waves, and this direction remained unchanged across EBIC γ values. This indicates a consistent difference in the overall magnitude of symptom connectivity between severity-defined profiles, depending on the specific classification examined. However, the omnibus network-structure test was significant only at Time 1, not at Time 2. The present findings therefore do not demonstrate stable qualitative differences in complete network configuration across waves. Node-level tests identify where some of the connectivity difference was concentrated, but exact centrality ranks remain descriptive and should not be interpreted as evidence of causal importance or as identifying optimal intervention targets (Bringmann et al., 2019; Dablander & Hinne, 2019).

The profile-specific centrality patterns observed in this study suggest that the conditional associations among symptoms differ across severity levels. The strength of centrality in cross-sectional networks should be interpreted with caution. This finding is consistent with network theory’s proposal that central symptoms may activate and sustain other symptoms (Fried et al., 2017; Fried & Cramer, 2017). As a conceptual suggestion rather than an empirical conclusion, it is possible that the symptoms that might occupy such a role depend on an individual’s position within the severity distribution. However, this interpretation would require longitudinal or experimental evidence for testing. Moreover, the lower-severity profile exhibited a denser network with more edges than the higher-severity profile, suggesting that, at lower severity levels, symptoms are more tightly interconnected, whereas at higher severity, the system may be more loosely organized with fewer but stronger pathways. This pattern was robust across the EBIC γ values examined. This finding is consistent with research showing that the predictability of individual symptoms from their neighbors varies across psychopathological networks (Haslbeck & Fried, 2017). The longitudinal transition data add a further layer of complexity to these findings.

The transition data suggest that profile membership is probabilistic rather than fixed. Approximately 57% of adolescents in the lower-severity profile and 62% in the higher-severity profile remained in the same profile across the four-year interval, indicating moderate temporal stability. This level of stability is consistent with prior longitudinal research on mental health profiles in youth, which has shown that, while subgroup membership is relatively stable, a substantial proportion of individuals transition between profiles over time (Moore et al., 2019). The significant chi-square test and Cramér’s *V* of 0.182 indicate that profile transitions are not random but follow a systematic pattern, albeit with considerable individual variability. Notably, the difference tests revealed that the four transition groups (stable lower, lower-to-higher, higher-to-lower, stable higher) differed primarily in between-profile contrasts rather than within-origin contrasts, suggesting that the distinction between lower-severity and higher-severity profiles is more consequential than whether an individual transitioned or remained stable. These findings carry theoretical and methodological implications for understanding the dynamic nature of psychopathology.

The difference in overall connectivity between the severity-defined profiles observed in this study contributes to the ongoing p-factor debate by extending it from the factor level to the symptom network level. The p-factor debate has largely been conducted using structural equation modeling and factor analysis, where the central question is whether a higher-order factor adequately captures the covariance among disorders (Caspi & Moffitt, 2018; Fried et al., 2021). Our findings suggest that even when a severity-based factor structure is supported, as evidenced by the two profiles differing primarily in overall symptom levels, the profiles differed reliably in overall symptom connectivity, whereas evidence for broader network-structure differences was wave-specific and should not be viewed as adjudicating between the p-factor and network frameworks. This implies that the p-factor captures how much psychopathology is present but not how that psychopathology is organized at the symptom level. Network theory (Borsboom, 2017) and the p-factor framework are not necessarily contradictory; rather, they capture different aspects of psychopathology’s structure. The p-factor describes the shared variance among disorders, whereas network analysis describes the pattern of conditional associations among symptoms within and across disorders.

Several limitations constrain these conclusions. First, item coverage is limited. The 14-item measure captures broad internalizing and externalizing dimensions but does not assess specific symptoms such as insomnia, appetite changes, or substance use, which may be central in adolescent psychopathology networks. Second, within-profile sample sizes, ranging from approximately 550 to 650 per profile, are at the lower end for stable network estimation, particularly for detecting small edge weights with precision (Epskamp & Fried, 2018). Although we used regularized estimation and evaluated stability via case-dropping bootstrap, the possibility of spurious edges or missed connections cannot be excluded. Third, the four-year interval between waves and the cross-sectional nature of the network estimates preclude causal inference about whether symptom activation cascades through the network as network theory predicts. Fourth, the sample is drawn from a single country (China) and lacks clinical validation. Participants were not diagnosed with clinical disorders, and whether the observed profiles correspond to clinically meaningful severity levels is unknown. Fifth, age and sex were not included as covariates in the latent profile or network models. This study’s focus was on describing and comparing symptom architecture across severity profiles derived from empirical data, and sex was balanced across waves. However, since symptom severity and network structure can change during adolescence, future studies should investigate whether these profiles and their networks vary by age and sex.

Finally, the profiles were derived from the same 14 symptoms that were subsequently used to estimate and compare the networks. However, conditioning on groups defined by these symptoms can induce range restriction and alter within-group covariance structures. Therefore, the observed differences in global strength may partly reflect the classification procedure rather than genuine differences in symptom connectivity (Boot et al., 2025; De Ron et al., 2021). Our inference is descriptive in that we describe the statistical networks conditional on the LPA-derived classification, rather than claiming to identify genuine underlying heterogeneity in symptom organization. Under this descriptive approach, conditioning on symptom-based groups is not necessarily problematic (Haslbeck et al., 2021). The two-profile solution provides a rough partition of severity rather than evidence that the population contains exactly two distinct subgroups, and the resulting conditional associations require replication in independent samples. These limitations suggest directions for future work.

The findings are primarily descriptive and hypothesis-generating. Recurring high-strength symptoms may be useful candidates for future longitudinal or experimental investigation, but cross-sectional centrality cannot establish that a symptom causes or maintains other symptoms, or that targeting it will improve outcomes. Likewise, because the present profiles differed primarily in severity, the study does not provide evidence for network-informed subtypes among adolescents with equivalent symptom severity. Future research could match individuals on total severity or model network heterogeneity conditional on severity and then test candidate targets in longitudinal or intervention designs.

## Figures and Tables

**Table 1:** Correspondence between abbreviations and specific items.

Abbreviations	Items
1. I1	I get upset when I run into difficulties with my studies
2. E1	I often get into arguments with my peers
3. I2	I’m afraid of exams
4. E2	I have a hard time concentrating
5. I3	I often feel lonely
6. E3	I get distracted easily
7. I4	I often feel sad and upset
8. E4	I’m having a hard time finishing my schoolwork.
9. I5	I’m worried that I’m not doing well enough in school
10. I6	I’m worried I won’t be able to finish my homework.
11. I7	I’m worried I won’t have any friends at school.
12. E5	I got into trouble for talking too much and bothering others.
13. E6	I got into trouble for fighting with kids my age.
14. I8	I felt ashamed when I made a mistake at school.

Note. Correspondence between item abbreviations and specific item contents for the internalizing and externalizing problem items.

**Table 2:** Descriptive statistics for internalizing and externalizing problem items at Time 1 and Time 2.

Table 2 Items	Time 1			Time 2		
	*M* [95 CI]	*SD*	*W*	*M* [95 CI]	*SD*	*W*
I1	2.46 [2.40, 2.53]	1.11	0.87	2.65 [2.59, 2.72]	1.19	0.82
E1	1.90 [1.85, 1.96]	0.97	0.78	1.73 [1.68, 1.78]	0.90	0.71
I2	2.33 [2.27, 2.40]	1.20	0.85	2.42 [2.35, 2.49]	1.28	0.83
E2	2.58 [2.51, 2.64]	1.17	0.87	2.61 [2.54, 2.68]	1.23	0.83
I3	1.97 [1.91, 2.03]	1.11	0.79	1.92 [1.86, 1.97]	1.03	0.75
E3	2.68 [2.61, 2.74]	1.20	0.88	2.72 [2.65, 2.79]	1.24	0.83
I4	1.85 [1.80, 1.91]	1.00	0.76	1.84 [1.79, 1.90]	0.98	0.74
E4	1.91 [1.85, 1.97]	1.09	0.77	1.88 [1.82, 1.94]	1.01	0.74
I5	2.73 [2.67, 2.80]	1.22	0.89	2.55 [2.48, 2.62]	1.27	0.83
I6	2.31 [2.23, 2.38]	1.29	0.83	2.33 [2.26, 2.41]	1.28	0.82
I7	1.89 [1.82, 1.95]	1.15	0.74	1.87 [1.80, 1.93]	1.09	0.73
E5	1.90 [1.84, 1.96]	1.02	0.78	1.90 [1.84, 1.96]	1.04	0.75
E6	1.63 [1.58, 1.68]	0.92	0.69	1.45 [1.41, 1.50]	0.79	0.60
I8	3.45 [3.38, 3.52]	1.27	0.86	3.49 [3.42, 3.56]	1.24	0.81

Note. Descriptive statistics (*M*, *SD*, 95% CI) and Shapiro–Wilk normality test results (*W*) for 14 items (eight internalizing [I1–I8], six externalizing [E1–E6]) assessed on a 5-point scale (1 = *completely inconsistent*, 5 = *completely compliant*). *N* = 1200. Items are derived from the Child Behavior Checklist as implemented in the CFPS. All Shapiro–Wilk tests were significant (*p* < 0.001), indicating departures from normality.

**Table 3:** Latent profile analysis fit indices at Time 1 and Time 2.

Classes	AIC	BIC	aBIC	Entropy	Minimum Variance	Solution Status
**Time 1**						
1	51,443.79	51,586.31	51,497.37			
2	48,005.24	48,295.38	48,114.32	0.8579	0.199	Retained
3	36,929.30	37,367.05	37,093.88	0.9853	0.000001	Boundary
4	30,630.32	31,215.68	30,850.39	0.9485	0.000001	Boundary
5	28,423.14	29,156.12	28,698.72	0.9766	0.000001	Boundary
**Time 2**						
1	50,960.15	51,102.67	51,013.73			
2	47,057.89	47,348.02	47,166.97	0.8693	0.190	Retained
3	30,977.83	31,415.58	31,142.41	0.9886	0.000001	Boundary
4	26,042.43	26,627.78	26,262.50	0.9968	0.000001	Boundary
5	20,612.41	21,345.38	20,887.98	0.9959	0.000001	Boundary

Note. Fit indices for one- to five-class solutions at Time 1 and Time 2. AIC = Akaike Information Criterion; BIC = Bayesian Information Criterion; aBIC = sample-size-adjusted BIC; Entropy = classification quality. Minimum variance is the smallest class-specific item variance. Values at the numerical lower bound (approximately 1 × 10^−6^) indicate a boundary or singular solution. Boldface identifies the solution retained after considering numerical admissibility, interpretability, cross-wave comparability, class size, and suitability for within-profile network estimation; it does not indicate that this solution minimized all information criteria.

**Table 4:** Descriptive statistics for internalizing and externalizing problem items by profile and wave.

Table 2 Items	Time 1				Time 2			
	Lower-Severity (*n* = 552)		Higher-Severity (*n* = 648)		Lower-Severity (*n* = 561)		Higher-Severity (*n* = 639)	
	*M* (*SD*) [95 CI]	*W*	*M* (*SD*) [95 CI]	*W*	*M* (*SD*) [95 CI]	*W*	*M* (*SD*) [95 CI]	*W*
I1	2.13 (0.96) [2.05, 2.21]	0.83	2.75 (1.14) [2.66, 2.84]	0.89	2.29 (1.08) [2.20, 2.37]	0.79	2.98 (1.19) [2.89, 3.07]	0.82
E1	1.49 (0.57) [1.45, 1.54]	0.70	2.25 (1.10) [2.16, 2.33]	0.84	1.41 (0.50) [1.37, 1.46]	0.64	2.01 (1.07) [1.93, 2.10]	0.77
I2	1.90 (0.99) [1.81, 1.98]	0.79	2.71 (1.24) [2.61, 2.80]	0.88	1.90 (1.02) [1.81, 1.98]	0.75	2.88 (1.30) [2.78, 2.98]	0.85
E2	2.12 (1.02) [2.03, 2.20]	0.82	2.97 (1.16) [2.88, 3.06]	0.88	2.07 (1.02) [1.99, 2.16]	0.77	3.08 (1.20) [2.99, 3.17]	0.83
I3	1.45 (0.56) [1.40, 1.50]	0.68	2.42 (1.26) [2.32, 2.51]	0.86	1.44 (0.50) [1.40, 1.48]	0.65	2.33 (1.18) [2.24, 2.43]	0.81
E3	2.24 (1.08) [2.15, 2.33]	0.85	3.04 (1.17) [2.95, 3.14]	0.89	2.24 (1.09) [2.15, 2.33]	0.79	3.14 (1.20) [3.05, 3.24]	0.82
I4	1.49 (0.61) [1.44, 1.55]	0.69	2.16 (1.15) [2.07, 2.25]	0.82	1.41 (0.50) [1.37, 1.45]	0.64	2.23 (1.13) [2.14, 2.32]	0.80
E4	1.39 (0.53) [1.35, 1.44]	0.65	2.35 (1.24) [2.25, 2.44]	0.86	1.42 (0.51) [1.37, 1.46]	0.64	2.29 (1.16) [2.20, 2.38]	0.81
I5	2.23 (1.10) [2.13, 2.32]	0.84	3.17 (1.15) [3.08, 3.26]	0.89	1.98 (1.00) [1.90, 2.06]	0.76	3.05 (1.27) [2.95, 3.15]	0.84
I6	1.66 (0.89) [1.59, 1.74]	0.71	2.85 (1.33) [2.75, 2.95]	0.88	1.64 (0.76) [1.57, 1.70]	0.70	2.95 (1.34) [2.84, 3.05]	0.84
I7	1.30 (0.46) [1.27, 1.34]	0.58	2.38 (1.32) [2.28, 2.48]	0.84	1.35 (0.48) [1.31, 1.39]	0.61	2.32 (1.27) [2.22, 2.42]	0.82
E5	1.47 (0.60) [1.42, 1.52]	0.69	2.27 (1.15) [2.18, 2.35]	0.85	1.46 (0.56) [1.41, 1.50]	0.65	2.30 (1.19) [2.20, 2.39]	0.81
E6	1.26 (0.44) [1.22, 1.30]	0.55	1.94 (1.09) [1.86, 2.02]	0.79	1.25 (0.44) [1.21, 1.28]	0.54	1.64 (0.97) [1.56, 1.71]	0.67
I8	3.30 (1.32) [3.19, 3.41]	0.87	3.58 (1.21) [3.49, 3.68]	0.85	3.23 (1.32) [3.12, 3.34]	0.83	3.73 (1.10) [3.64, 3.81]	0.77

Note. Descriptive statistics (*M*, *SD*, 95% CI) and Shapiro–Wilk normality test results (*W*) for 14 items (eight internalizing [I1–I8], six externalizing [E1–E6]) assessed on a 5-point scale (1 = *completely inconsistent*, 5 = *completely compliant*). Lower-severity and higher-severity profiles were identified via latent profile analysis at each wave. *N* = 1200. Items are derived from the Child Behavior Checklist as implemented in the CFPS. All Shapiro–Wilk tests were significant (*p* < 0.001), indicating departures from normality.

**Table 5:** Row-standardized profile transition probabilities (Time 1 to Time 2).

	To Time 2 Lower-Severity	To Time 2 Higher-Severity
From Time 1 Lower-Severity	56.70	43.30
From Time 1 Higher-Severity	38.27	61.73

Note. Row-standardized transition probabilities (%) from Time 1 profile membership to Time 2 profile membership. Lower-severity profile at Time 1 (*n* = 552, 46.0%) and Time 2 (*n* = 561, 46.8%). Higher-severity profile at Time 1 (*n* = 648, 54.0%) and Time 2 (*n* = 639, 53.3%). Values represent the percentage of adolescents in each Time 1 profile who transitioned to each Time 2 profile.

**Table 6:** Kruskal–Wallis *H* tests across transition groups.

Items	Time 1			Time 2		
	*H*(3)	*p*	ε^2^	*H*(3)	*p*	ε^2^
I1	94.47	<0.001	0.08	100.36	<0.001	0.08
E1	162.62	<0.001	0.13	114.24	<0.001	0.09
I2	138.56	<0.001	0.11	174.62	<0.001	0.14
E2	162.96	<0.001	0.13	212.81	<0.001	0.18
I3	204.94	<0.001	0.17	215.26	<0.001	0.18
E3	138.19	<0.001	0.11	164.81	<0.001	0.14
I4	118.38	<0.001	0.10	194.89	<0.001	0.16
E4	214.52	<0.001	0.18	227.90	<0.001	0.19
I5	187.30	<0.001	0.15	202.96	<0.001	0.17
I6	253.73	<0.001	0.21	284.73	<0.001	0.24
I7	250.82	<0.001	0.21	220.89	<0.001	0.18
E5	165.13	<0.001	0.14	178.67	<0.001	0.15
E6	148.33	<0.001	0.12	59.27	<0.001	0.05
I8	14.12	0.003	0.01	43.46	<0.001	0.03

Note. Kruskal–Wallis *H* tests comparing item scores across four transition groups (stable lower profile, lower-to-higher, higher-to-lower, stable higher profile) at both Time 1 and Time 2.

**Table 7:** Network centrality (strength) by profile and wave.

Items	Time 1				Time 2			
	Lower-Severity		Higher-Severity		Lower-Severity		Higher-Severity	
	Strength	95% CI	Strength	95% CI	Strength	95% CI	Strength	95% CI
I1	0.00	[0.00, 0.11]	0.06	[0.00, 0.23]	0.09	[0.00, 0.38]	0.23	[0.11, 0.42]
E1	0.19	[0.11, 0.43]	0.34	[0.16, 0.57]	0.39	[0.31, 0.65]	0.24	[0.10, 0.48]
I2	0.00	[0.00, 0.12]	0.10	[0.05, 0.42]	0.29	[0.16, 0.52]	0.42	[0.25, 0.62]
E2	0.30	[0.22, 0.46]	0.27	[0.17, 0.48]	0.42	[0.32, 0.63]	0.33	[0.22, 0.47]
I3	0.44	[0.25, 0.63]	0.17	[0.10, 0.35]	0.71	[0.56, 0.90]	0.36	[0.24, 0.59]
E3	0.30	[0.24, 0.52]	0.27	[0.20, 0.56]	0.39	[0.29, 0.59]	0.37	[0.27, 0.56]
I4	0.31	[0.14, 0.53]	0.25	[0.15, 0.51]	0.93	[0.78, 1.10]	0.33	[0.22, 0.59]
E4	0.36	[0.22, 0.67]	0.27	[0.12, 0.51]	0.85	[0.60, 1.05]	0.00	[0.00, 0.14]
I5	0.19	[0.06, 0.36]	0.31	[0.23, 0.56]	0.41	[0.25, 0.62]	0.52	[0.34, 0.70]
I6	0.25	[0.14, 0.44]	0.41	[0.29, 0.64]	0.48	[0.35, 0.66]	0.42	[0.27, 0.57]
I7	0.86	[0.67, 1.04]	0.09	[0.00, 0.23]	1.05	[0.91, 1.22]	0.27	[0.12, 0.53]
E5	0.39	[0.27, 0.58]	0.28	[0.18, 0.54]	0.55	[0.40, 0.85]	0.12	[0.00, 0.35]
E6	0.77	[0.58, 0.95]	0.38	[0.26, 0.54]	0.62	[0.48, 0.80]	0.25	[0.12, 0.46]
I8	0.06	[0.00, 0.22]	0.12	[0.05, 0.33]	0.08	[0.00, 0.25]	0.11	[0.00, 0.28]

Note. Strength centrality values from Gaussian graphical models estimated via Graphical LASSO with EBIC model selection (gamma = 0.5). Total of 14 nodes (eight internalizing [I1–I8], six externalizing [E1–E6]). 1000 bootstrap replications. Strength centrality is stable across all four profiles (CS > 0.70). Betweenness and closeness centrality are reported in the Supplementary Materials due to instability (CS < 0.50 for most profiles). Higher values indicate greater connectivity within the network.

## Data Availability

The dataset used in this study was obtained from China Family Panel Studies (https://doi.org/10.18170/DVN/45LCSO).

## Supplementary Materials

The following supporting information can be downloaded at: https://www.mdpi.com/article/10.3390/bs16091643/s1.
