# Supplementary material for: Lower Severity, Denser Network: Profile-Specific Symptom Architecture in Adolescent Internalizing and Externalizing Problems
Source: Behav Sci (Basel). 2026 Sep 14;16(9):1643. doi: 10.3390/bs16091643 (PMC13603138; doi:10.3390/bs16091643)
Supplement: Supplementary file 1 [file behavsci-16-01643-s001.zip › behavsci-4512497-supplementary.pdf]

Supplementary Materials:

Lower Severity, Denser Network: Profile-specific Symptom Architecture in Adolescent Internalizing and Externalizing Problems

Sample Selection and Attrition. The analytic sample was drawn from the China Family Panel Studies child questionnaire. At Time 1 in 2018, 2,478 children aged 9 to 15 years completed the 14 items assessing internalizing and externalizing problems. At Time 2 in 2022, 2,872 respondents completed the same items. Matching participants across the two waves by their unique personal identifier produced 1,200 adolescents who provided data at both waves, and these participants formed the analytic sample. The remaining participants included 1,278 who provided data at Time 1 only and 1,672 who provided data at Time 2 only. To assess whether panel attrition introduced selection bias, we compared the 1,200 retained adolescents with the 1,278 who participated only at Time 1 on sex, age, and total symptom severity measured at Time 1. The retained and attrited groups did not differ significantly on sex, with 53.8% male in the retained group and 53.2% male in the attrited group, $\chi^2(1) = 0.10$, $p = .755$. The retained group was slightly younger than the attrited group, $M = 12.12$ ($SD = 1.55$) versus $M = 12.62$ ($SD = 1.75$), $t(2467) = -7.54$, $p < .001$. The two groups did not differ significantly on total symptom severity, $M = 31.59$ ($SD = 8.10$) versus $M = 32.07$ ($SD = 8.19$), $t(2467) = -1.46$, $p = .145$, a comparison based on 1,200 and 1,275 participants after excluding three attrited participants with missing item responses. One participant had a single missing item response at Time 2, which was imputed with the item mean, and no participants were excluded for missing data.

Table S1. Correlation Matrices at Time 1

Variable	1	2	3	4	5	6	7	8	9	10	11	12	13	14
1.I1	—													
2.E1	0.25***	—												
3.I2	0.18***	0.24***	—											
4.E2	0.17***	0.24***	0.26***	—										
5.I3	0.15***	0.26***	0.23***	0.21***	—									
6.E3	0.16***	0.23***	0.23***	0.45***	0.22***	—								
7.I4	0.13***	0.26***	0.22***	0.15***	0.42***	0.18***	—							
8.E4	0.17***	0.28***	0.29***	0.30***	0.33***	0.25***	0.34***	—						
9.I5	0.16***	0.15***	0.23***	0.21***	0.22***	0.26***	0.19***	0.18***	—					
10.I6	0.19***	0.26***	0.29***	0.28***	0.25***	0.27***	0.26***	0.42***	0.40***	—				
11.I7	0.14***	0.24***	0.22***	0.17***	0.34***	0.14***	0.35***	0.33***	0.27***	0.41***	—			
12.E5	0.19***	0.35***	0.20***	0.17***	0.30***	0.18***	0.28***	0.31***	0.19***	0.26***	0.31***	—		
13.E6	0.13***	0.39***	0.21***	0.18***	0.26***	0.17***	0.26***	0.35***	0.16***	0.24***	0.29***	0.48***	—	
14.I8	0.08**	0.02	0.09**	0.07*	0.08**	0.13***	0.03	-0.02	0.22***	0.13***	0.07*	0.07*	0.03	—

Note. Spearman correlation matrices for 14 items at Time 1. $N = 1{,}200$. Items I1–I8 measure internalizing problems; items E1–E6 measure externalizing problems. $^{*}p < .05$ $^{**}p < .01$ $^{***}p < .001$

Table S2. Correlation Matrices at Time 2

Variable	1	2	3	4	5	6	7	8	9	10	11	12	13	14
1.I1	—													
2.E1	0.27***	—												
3.I2	0.32***	0.27***	—											
4.E2	0.23***	0.22***	0.31***	—										
5.I3	0.21***	0.35***	0.29***	0.26***	—									
6.E3	0.22***	0.22***	0.27***	0.50***	0.28***	—								
7.I4	0.20***	0.34***	0.32***	0.23***	0.54***	0.32***	—							
8.E4	0.18***	0.27***	0.27***	0.33***	0.30***	0.27***	0.34***	—						
9.I5	0.26***	0.20***	0.39***	0.21***	0.33***	0.24***	0.30***	0.26***	—					
10.I6	0.27***	0.24***	0.41***	0.27***	0.33***	0.26***	0.33***	0.39***	0.50***	—				
11.I7	0.19***	0.32***	0.35***	0.26***	0.48***	0.24***	0.44***	0.37***	0.36***	0.48***	—			
12.E5	0.20***	0.29***	0.28***	0.25***	0.32***	0.26***	0.37***	0.35***	0.31***	0.35***	0.43***	—		
13.E6	0.12***	0.34***	0.22***	0.15***	0.28***	0.13***	0.28***	0.32***	0.13***	0.22***	0.36***	0.40***	—	
14.I8	0.14***	-0.04	0.11***	0.08**	0.02	0.12***	0.04	-0.03	0.23***	0.15***	0.10***	0.09**	-0.05	—

Note. Spearman correlation matrices for 14 items at Time 2. $N = 1{,}200$. Items I1–I8 measure internalizing problems; items E1–E6 measure externalizing problems. ** $p < .01$ *** $p < .001$

Table S3

Post-hoc analysis of the results of the Kruskal-Wallis H test for the scores of the four transition groups.

Variable	Group1	Group2	Statistic	Test	MeanRank1	MeanRank2	p_adjusted	significant	effect_size	effect_size_type
I1	T1_P1→T2_P1	T1_P1→T2_P2	0.21	Dunn'sz	503.41	497.55	1.000	No	0.00	rank-eta²
I1	T1_P1→T2_P1	T1_P2→T2_P1	-7.06	Dunn'sz	503.41	700.98	<.001	Yes	0.04	rank-eta²
I1	T1_P1→T2_P1	T1_P2→T2_P2	-6.93	Dunn'sz	503.41	675.69	<.001	Yes	0.04	rank-eta²
I1	T1_P1→T2_P2	T1_P2→T2_P1	-6.81	Dunn'sz	497.55	700.98	<.001	Yes	0.04	rank-eta²
I1	T1_P1→T2_P2	T1_P2→T2_P2	-6.62	Dunn'sz	497.55	675.69	<.001	Yes	0.04	rank-eta²
I1	T1_P2→T2_P1	T1_P2→T2_P2	0.95	Dunn'sz	700.98	675.69	1.000	No	0.00	rank-eta²
E1	T1_P1→T2_P1	T1_P1→T2_P2	0.02	Dunn'sz	473.06	472.38	1.000	No	0.00	rank-eta²
E1	T1_P1→T2_P1	T1_P2→T2_P1	-8.73	Dunn'sz	473.06	710.87	<.001	Yes	0.06	rank-eta²
E1	T1_P1→T2_P1	T1_P2→T2_P2	-9.73	Dunn'sz	473.06	708.34	<.001	Yes	0.08	rank-eta²
E1	T1_P1→T2_P2	T1_P2→T2_P1	-8.22	Dunn'sz	472.38	710.87	<.001	Yes	0.06	rank-eta²
E1	T1_P1→T2_P2	T1_P2→T2_P2	-9.01	Dunn'sz	472.38	708.34	<.001	Yes	0.07	rank-eta²
E1	T1_P2→T2_P1	T1_P2→T2_P2	0.10	Dunn'sz	710.87	708.34	1.000	No	0.00	rank-eta²
I2	T1_P1→T2_P1	T1_P1→T2_P2	-0.89	Dunn'sz	467.73	493.18	1.000	No	0.00	rank-eta²
I2	T1_P1→T2_P1	T1_P2→T2_P1	-8.50	Dunn'sz	467.73	707.45	<.001	Yes	0.06	rank-eta²
I2	T1_P1→T2_P1	T1_P2→T2_P2	-9.37	Dunn'sz	467.73	702.21	<.001	Yes	0.07	rank-eta²
I2	T1_P1→T2_P2	T1_P2→T2_P1	-7.13	Dunn'sz	493.18	707.45	<.001	Yes	0.04	rank-eta²
I2	T1_P1→T2_P2	T1_P2→T2_P2	-7.71	Dunn'sz	493.18	702.21	<.001	Yes	0.05	rank-eta²
I2	T1_P2→T2_P1	T1_P2→T2_P2	0.20	Dunn'sz	707.45	702.21	1.000	No	0.00	rank-eta²
E2	T1_P1→T2_P1	T1_P1→T2_P2	-1.25	Dunn'sz	453.83	489.35	1.000	No	0.00	rank-eta²
E2	T1_P1→T2_P1	T1_P2→T2_P1	-9.49	Dunn'sz	453.83	720.52	<.001	Yes	0.08	rank-eta²
E2	T1_P1→T2_P1	T1_P2→T2_P2	-10.16	Dunn'sz	453.83	707.27	<.001	Yes	0.09	rank-eta²
E2	T1_P1→T2_P2	T1_P2→T2_P1	-7.71	Dunn'sz	489.35	720.52	<.001	Yes	0.05	rank-eta²

Variable	Group1	Group2	Statistic	Test	MeanRank1	MeanRank2	p_adjusted	significant	effect_size	effect_size_type
E2	T1_P1→T2_P2	T1_P2→T2_P2	-8.06	Dunn'sz	489.35	707.27	<.001	Yes	0.05	rank-eta²
E2	T1_P2→T2_P1	T1_P2→T2_P2	0.50	Dunn'sz	720.52	707.27	1.000	No	0.00	rank-eta²
I3	T1_P1→T2_P1	T1_P1→T2_P2	-0.10	Dunn'sz	454.59	457.27	1.000	No	0.00	rank-eta²
I3	T1_P1→T2_P1	T1_P2→T2_P1	-9.13	Dunn'sz	454.59	706.19	<.001	Yes	0.07	rank-eta²
I3	T1_P1→T2_P1	T1_P2→T2_P2	-11.45	Dunn'sz	454.59	734.72	<.001	Yes	0.11	rank-eta²
I3	T1_P1→T2_P2	T1_P2→T2_P1	-8.47	Dunn'sz	457.27	706.19	<.001	Yes	0.06	rank-eta²
I3	T1_P1→T2_P2	T1_P2→T2_P2	-10.47	Dunn'sz	457.27	734.72	<.001	Yes	0.09	rank-eta²
I3	T1_P2→T2_P1	T1_P2→T2_P2	-1.09	Dunn'sz	706.19	734.72	1.000	No	0.00	rank-eta²
E3	T1_P1→T2_P1	T1_P1→T2_P2	-0.64	Dunn'sz	470.98	489.23	1.000	No	0.00	rank-eta²
E3	T1_P1→T2_P1	T1_P2→T2_P1	-7.44	Dunn'sz	470.98	681.84	<.001	Yes	0.05	rank-eta²
E3	T1_P1→T2_P1	T1_P2→T2_P2	-9.81	Dunn'sz	470.98	717.90	<.001	Yes	0.08	rank-eta²
E3	T1_P1→T2_P2	T1_P2→T2_P1	-6.37	Dunn'sz	489.23	681.84	<.001	Yes	0.03	rank-eta²
E3	T1_P1→T2_P2	T1_P2→T2_P2	-8.39	Dunn'sz	489.23	717.90	<.001	Yes	0.06	rank-eta²
E3	T1_P2→T2_P1	T1_P2→T2_P2	-1.34	Dunn'sz	681.84	717.90	1.000	No	0.00	rank-eta²
I4	T1_P1→T2_P1	T1_P1→T2_P2	-1.95	Dunn'sz	471.49	525.17	.304	No	0.00	rank-eta²
I4	T1_P1→T2_P1	T1_P2→T2_P1	-7.10	Dunn'sz	471.49	664.40	<.001	Yes	0.04	rank-eta²
I4	T1_P1→T2_P1	T1_P2→T2_P2	-9.75	Dunn'sz	471.49	706.84	<.001	Yes	0.08	rank-eta²
I4	T1_P1→T2_P2	T1_P2→T2_P1	-4.80	Dunn'sz	525.17	664.40	<.001	Yes	0.02	rank-eta²
I4	T1_P1→T2_P2	T1_P2→T2_P2	-6.95	Dunn'sz	525.17	706.84	<.001	Yes	0.04	rank-eta²
I4	T1_P2→T2_P1	T1_P2→T2_P2	-1.64	Dunn'sz	664.40	706.84	.603	No	0.00	rank-eta²
E4	T1_P1→T2_P1	T1_P1→T2_P2	-1.41	Dunn'sz	436.91	475.93	.953	No	0.00	rank-eta²
E4	T1_P1→T2_P1	T1_P2→T2_P1	-9.99	Dunn'sz	436.91	710.73	<.001	Yes	0.08	rank-eta²
E4	T1_P1→T2_P1	T1_P2→T2_P2	-12.24	Dunn'sz	436.91	734.60	<.001	Yes	0.12	rank-eta²
E4	T1_P1→T2_P2	T1_P2→T2_P1	-8.04	Dunn'sz	475.93	710.73	<.001	Yes	0.05	rank-eta²
E4	T1_P1→T2_P2	T1_P2→T2_P2	-9.81	Dunn'sz	475.93	734.60	<.001	Yes	0.08	rank-eta²

Variable	Group1	Group2	Statistic	Test	MeanRank1	MeanRank2	p_adjusted	significant	effect_size	effect_size_type
E4	T1_P2→T2_P1	T1_P2→T2_P2	-0.92	Dunn'sz	710.73	734.60	1.000	No	0.00	rank-eta²
I5	T1_P1→T2_P1	T1_P1→T2_P2	-0.20	Dunn'sz	457.08	462.75	1.000	No	0.00	rank-eta²
I5	T1_P1→T2_P1	T1_P2→T2_P1	-7.84	Dunn'sz	457.08	680.07	<.001	Yes	0.05	rank-eta²
I5	T1_P1→T2_P1	T1_P2→T2_P2	-11.43	Dunn'sz	457.08	745.70	<.001	Yes	0.11	rank-eta²
I5	T1_P1→T2_P2	T1_P2→T2_P1	-7.16	Dunn'sz	462.75	680.07	<.001	Yes	0.04	rank-eta²
I5	T1_P1→T2_P2	T1_P2→T2_P2	-10.34	Dunn'sz	462.75	745.70	<.001	Yes	0.09	rank-eta²
I5	T1_P2→T2_P1	T1_P2→T2_P2	-2.43	Dunn'sz	680.07	745.70	.091	No	0.00	rank-eta²
I6	T1_P1→T2_P1	T1_P1→T2_P2	0.06	Dunn'sz	437.22	435.52	1.000	No	0.00	rank-eta²
I6	T1_P1→T2_P1	T1_P2→T2_P1	-9.50	Dunn'sz	437.22	705.51	<.001	Yes	0.08	rank-eta²
I6	T1_P1→T2_P1	T1_P2→T2_P2	-12.95	Dunn'sz	437.22	761.73	<.001	Yes	0.14	rank-eta²
I6	T1_P1→T2_P2	T1_P2→T2_P1	-8.97	Dunn'sz	435.52	705.51	<.001	Yes	0.07	rank-eta²
I6	T1_P1→T2_P2	T1_P2→T2_P2	-12.01	Dunn'sz	435.52	761.73	<.001	Yes	0.12	rank-eta²
I6	T1_P2→T2_P1	T1_P2→T2_P2	-2.09	Dunn'sz	705.51	761.73	.217	No	0.00	rank-eta²
I7	T1_P1→T2_P1	T1_P1→T2_P2	-0.33	Dunn'sz	440.45	449.38	1.000	No	0.00	rank-eta²
I7	T1_P1→T2_P1	T1_P2→T2_P1	-9.41	Dunn'sz	440.45	695.67	<.001	Yes	0.07	rank-eta²
I7	T1_P1→T2_P1	T1_P2→T2_P2	-13.15	Dunn'sz	440.45	757.02	<.001	Yes	0.14	rank-eta²
I7	T1_P1→T2_P2	T1_P2→T2_P1	-8.52	Dunn'sz	449.38	695.67	<.001	Yes	0.06	rank-eta²
I7	T1_P1→T2_P2	T1_P2→T2_P2	-11.80	Dunn'sz	449.38	757.02	<.001	Yes	0.12	rank-eta²
I7	T1_P2→T2_P1	T1_P2→T2_P2	-2.38	Dunn'sz	695.67	757.02	.104	No	0.00	rank-eta²
E5	T1_P1→T2_P1	T1_P1→T2_P2	-0.31	Dunn'sz	468.98	477.63	1.000	No	0.00	rank-eta²
E5	T1_P1→T2_P1	T1_P2→T2_P1	-7.57	Dunn'sz	468.98	676.31	<.001	Yes	0.05	rank-eta²
E5	T1_P1→T2_P1	T1_P2→T2_P2	-10.73	Dunn'sz	468.98	729.83	<.001	Yes	0.10	rank-eta²
E5	T1_P1→T2_P2	T1_P2→T2_P1	-6.80	Dunn'sz	477.63	676.31	<.001	Yes	0.04	rank-eta²
E5	T1_P1→T2_P2	T1_P2→T2_P2	-9.58	Dunn'sz	477.63	729.83	<.001	Yes	0.08	rank-eta²
E5	T1_P2→T2_P1	T1_P2→T2_P2	-2.06	Dunn'sz	676.31	729.83	.239	No	0.00	rank-eta²

Variable	Group1	Group2	Statistic	Test	MeanRank1	MeanRank2	p_adjusted	significant	effect_size	effect_size_type
E6	T1_P1→T2_P1	T1_P1→T2_P2	-0.64	Dunn'sz	477.14	493.96	1.000	No	0.00	rank-eta^2
E6	T1_P1→T2_P1	T1_P2→T2_P1	-9.04	Dunn'sz	477.14	712.16	<.001	Yes	0.07	rank-eta^2
E6	T1_P1→T2_P1	T1_P2→T2_P2	-9.29	Dunn'sz	477.14	691.46	<.001	Yes	0.07	rank-eta^2
E6	T1_P1→T2_P2	T1_P2→T2_P1	-7.87	Dunn'sz	493.96	712.16	<.001	Yes	0.05	rank-eta^2
E6	T1_P1→T2_P2	T1_P2→T2_P2	-7.90	Dunn'sz	493.96	691.46	<.001	Yes	0.05	rank-eta^2
E6	T1_P2→T2_P1	T1_P2→T2_P2	0.84	Dunn'sz	712.16	691.46	1.000	No	0.00	rank-eta^2
I8	T1_P1→T2_P1	T1_P1→T2_P2	-0.37	Dunn'sz	558.30	568.99	1.000	No	0.00	rank-eta^2
I8	T1_P1→T2_P1	T1_P2→T2_P1	-2.09	Dunn'sz	558.30	617.15	.221	No	0.00	rank-eta^2
I8	T1_P1→T2_P1	T1_P2→T2_P2	-3.34	Dunn'sz	558.30	642.02	.005	Yes	0.01	rank-eta^2
I8	T1_P1→T2_P2	T1_P2→T2_P1	-1.60	Dunn'sz	568.99	617.15	.655	No	0.00	rank-eta^2
I8	T1_P1→T2_P2	T1_P2→T2_P2	-2.69	Dunn'sz	568.99	642.02	.042	Yes	0.01	rank-eta^2
I8	T1_P2→T2_P1	T1_P2→T2_P2	-0.93	Dunn'sz	617.15	642.02	1.000	No	0.00	rank-eta^2
I1_2022	T1_P1→T2_P1	T1_P1→T2_P2	-6.06	Dunn'sz	498.55	666.88	<.001	Yes	0.03	rank-eta^2
I1_2022	T1_P1→T2_P1	T1_P2→T2_P1	-0.25	Dunn'sz	498.55	505.30	1.000	No	0.00	rank-eta^2
I1_2022	T1_P1→T2_P1	T1_P2→T2_P2	-8.24	Dunn'sz	498.55	699.64	<.001	Yes	0.06	rank-eta^2
I1_2022	T1_P1→T2_P2	T1_P2→T2_P1	5.51	Dunn'sz	666.88	505.30	<.001	Yes	0.03	rank-eta^2
I1_2022	T1_P1→T2_P2	T1_P2→T2_P2	-1.24	Dunn'sz	666.88	699.64	1.000	No	0.00	rank-eta^2
I1_2022	T1_P2→T2_P1	T1_P2→T2_P2	-7.44	Dunn'sz	505.30	699.64	<.001	Yes	0.05	rank-eta^2
E1_2022	T1_P1→T2_P1	T1_P1→T2_P2	-7.91	Dunn'sz	468.10	680.93	<.001	Yes	0.05	rank-eta^2
E1_2022	T1_P1→T2_P1	T1_P2→T2_P1	-2.80	Dunn'sz	468.10	542.71	.030	Yes	0.01	rank-eta^2
E1_2022	T1_P1→T2_P1	T1_P2→T2_P2	-9.47	Dunn'sz	468.10	691.88	<.001	Yes	0.07	rank-eta^2
E1_2022	T1_P1→T2_P2	T1_P2→T2_P1	4.87	Dunn'sz	680.93	542.71	<.001	Yes	0.02	rank-eta^2
E1_2022	T1_P1→T2_P2	T1_P2→T2_P2	-0.43	Dunn'sz	680.93	691.88	1.000	No	0.00	rank-eta^2
E1_2022	T1_P2→T2_P1	T1_P2→T2_P2	-5.89	Dunn'sz	542.71	691.88	<.001	Yes	0.03	rank-eta^2
I2_2022	T1_P1→T2_P1	T1_P1→T2_P2	-8.66	Dunn'sz	463.79	708.83	<.001	Yes	0.06	rank-eta^2

Variable	Group1	Group2	Statistic	Test	MeanRank1	MeanRank2	p_adjusted	significant	effect_size	effect_size_type
I2_2022	T1_P1→T2_P1	T1_P2→T2_P1	-0.23	Dunn'sz	463.79	470.10	1.000	No	0.00	rank-eta²
I2_2022	T1_P1→T2_P1	T1_P2→T2_P2	-10.46	Dunn'sz	463.79	723.60	<.001	Yes	0.09	rank-eta²
I2_2022	T1_P1→T2_P2	T1_P2→T2_P1	8.00	Dunn'sz	708.83	470.10	<.001	Yes	0.05	rank-eta²
I2_2022	T1_P1→T2_P2	T1_P2→T2_P2	-0.55	Dunn'sz	708.83	723.60	1.000	No	0.00	rank-eta²
I2_2022	T1_P2→T2_P1	T1_P2→T2_P2	-9.53	Dunn'sz	470.10	723.60	<.001	Yes	0.08	rank-eta²
E2_2022	T1_P1→T2_P1	T1_P1→T2_P2	-8.12	Dunn'sz	446.98	674.28	<.001	Yes	0.05	rank-eta²
E2_2022	T1_P1→T2_P1	T1_P2→T2_P1	-0.87	Dunn'sz	446.98	470.99	1.000	No	0.00	rank-eta²
E2_2022	T1_P1→T2_P1	T1_P2→T2_P2	-12.60	Dunn'sz	446.98	756.84	<.001	Yes	0.13	rank-eta²
E2_2022	T1_P1→T2_P2	T1_P2→T2_P1	6.88	Dunn'sz	674.28	470.99	<.001	Yes	0.04	rank-eta²
E2_2022	T1_P1→T2_P2	T1_P2→T2_P2	-3.10	Dunn'sz	674.28	756.84	.012	Yes	0.01	rank-eta²
E2_2022	T1_P2→T2_P1	T1_P2→T2_P2	-10.85	Dunn'sz	470.99	756.84	<.001	Yes	0.10	rank-eta²
I3_2022	T1_P1→T2_P1	T1_P1→T2_P2	-8.21	Dunn'sz	447.16	671.56	<.001	Yes	0.06	rank-eta²
I3_2022	T1_P1→T2_P1	T1_P2→T2_P1	-1.12	Dunn'sz	447.16	477.37	1.000	No	0.00	rank-eta²
I3_2022	T1_P1→T2_P1	T1_P2→T2_P2	-12.79	Dunn'sz	447.16	754.37	<.001	Yes	0.14	rank-eta²
I3_2022	T1_P1→T2_P2	T1_P2→T2_P1	6.73	Dunn'sz	671.56	477.37	<.001	Yes	0.04	rank-eta²
I3_2022	T1_P1→T2_P2	T1_P2→T2_P2	-3.18	Dunn'sz	671.56	754.37	.009	Yes	0.01	rank-eta²
I3_2022	T1_P2→T2_P1	T1_P2→T2_P2	-10.77	Dunn'sz	477.37	754.37	<.001	Yes	0.10	rank-eta²
E3_2022	T1_P1→T2_P1	T1_P1→T2_P2	-8.32	Dunn'sz	447.56	680.43	<.001	Yes	0.06	rank-eta²
E3_2022	T1_P1→T2_P1	T1_P2→T2_P1	-2.24	Dunn'sz	447.56	509.51	.152	No	0.00	rank-eta²
E3_2022	T1_P1→T2_P1	T1_P2→T2_P2	-11.44	Dunn'sz	447.56	728.83	<.001	Yes	0.11	rank-eta²
E3_2022	T1_P1→T2_P2	T1_P2→T2_P1	5.79	Dunn'sz	680.43	509.51	<.001	Yes	0.03	rank-eta²
E3_2022	T1_P1→T2_P2	T1_P2→T2_P2	-1.82	Dunn'sz	680.43	728.83	.415	No	0.00	rank-eta²
E3_2022	T1_P2→T2_P1	T1_P2→T2_P2	-8.33	Dunn'sz	509.51	728.83	<.001	Yes	0.06	rank-eta²
I4_2022	T1_P1→T2_P1	T1_P1→T2_P2	-8.64	Dunn'sz	467.74	702.77	<.001	Yes	0.06	rank-eta²
I4_2022	T1_P1→T2_P1	T1_P2→T2_P1	0.26	Dunn'sz	467.74	460.79	1.000	No	0.00	rank-eta²

Variable	Group1	Group2	Statistic	Test	MeanRank1	MeanRank2	p_adjusted	significant	effect_size	effect_size_type
I4_2022	T1_P1→T2_P1	T1_P2→T2_P2	-10.97	Dunn'sz	467.74	729.89	<.001	Yes	0.10	rank-eta²
I4_2022	T1_P1→T2_P2	T1_P2→T2_P1	8.43	Dunn'sz	702.77	460.79	<.001	Yes	0.06	rank-eta²
I4_2022	T1_P1→T2_P2	T1_P2→T2_P2	-1.05	Dunn'sz	702.77	729.89	1.000	No	0.00	rank-eta²
I4_2022	T1_P2→T2_P1	T1_P2→T2_P2	-10.51	Dunn'sz	460.79	729.89	<.001	Yes	0.09	rank-eta²
E4_2022	T1_P1→T2_P1	T1_P1→T2_P2	-7.70	Dunn'sz	456.23	666.38	<.001	Yes	0.05	rank-eta²
E4_2022	T1_P1→T2_P1	T1_P2→T2_P1	-0.09	Dunn'sz	456.23	458.59	1.000	No	0.00	rank-eta²
E4_2022	T1_P1→T2_P1	T1_P2→T2_P2	-12.76	Dunn'sz	456.23	762.01	<.001	Yes	0.14	rank-eta²
E4_2022	T1_P1→T2_P2	T1_P2→T2_P1	7.22	Dunn'sz	666.38	458.59	<.001	Yes	0.04	rank-eta²
E4_2022	T1_P1→T2_P2	T1_P2→T2_P2	-3.68	Dunn'sz	666.38	762.01	.001	Yes	0.01	rank-eta²
E4_2022	T1_P2→T2_P1	T1_P2→T2_P2	-11.82	Dunn'sz	458.59	762.01	<.001	Yes	0.12	rank-eta²
I5_2022	T1_P1→T2_P1	T1_P1→T2_P2	-10.26	Dunn'sz	446.11	735.88	<.001	Yes	0.09	rank-eta²
I5_2022	T1_P1→T2_P1	T1_P2→T2_P1	-0.84	Dunn'sz	446.11	469.72	1.000	No	0.00	rank-eta²
I5_2022	T1_P1→T2_P1	T1_P2→T2_P2	-11.10	Dunn'sz	446.11	721.50	<.001	Yes	0.10	rank-eta²
I5_2022	T1_P1→T2_P2	T1_P2→T2_P1	8.93	Dunn'sz	735.88	469.72	<.001	Yes	0.07	rank-eta²
I5_2022	T1_P1→T2_P2	T1_P2→T2_P2	0.53	Dunn'sz	735.88	721.50	1.000	No	0.00	rank-eta²
I5_2022	T1_P2→T2_P1	T1_P2→T2_P2	-9.48	Dunn'sz	469.72	721.50	<.001	Yes	0.07	rank-eta²
I6_2022	T1_P1→T2_P1	T1_P1→T2_P2	-10.19	Dunn'sz	432.84	721.28	<.001	Yes	0.09	rank-eta²
I6_2022	T1_P1→T2_P1	T1_P2→T2_P1	0.22	Dunn'sz	432.84	426.57	1.000	No	0.00	rank-eta²
I6_2022	T1_P1→T2_P1	T1_P2→T2_P2	-13.45	Dunn'sz	432.84	767.36	<.001	Yes	0.15	rank-eta²
I6_2022	T1_P1→T2_P2	T1_P2→T2_P1	9.87	Dunn'sz	721.28	426.57	<.001	Yes	0.08	rank-eta²
I6_2022	T1_P1→T2_P2	T1_P2→T2_P2	-1.71	Dunn'sz	721.28	767.36	.523	No	0.00	rank-eta²
I6_2022	T1_P2→T2_P1	T1_P2→T2_P2	-12.79	Dunn'sz	426.57	767.36	<.001	Yes	0.14	rank-eta²
I7_2022	T1_P1→T2_P1	T1_P1→T2_P2	-8.81	Dunn'sz	451.81	692.86	<.001	Yes	0.06	rank-eta²
I7_2022	T1_P1→T2_P1	T1_P2→T2_P1	-0.37	Dunn'sz	451.81	461.77	1.000	No	0.00	rank-eta²
I7_2022	T1_P1→T2_P1	T1_P2→T2_P2	-12.31	Dunn'sz	451.81	747.67	<.001	Yes	0.13	rank-eta²

Variable	Group1	Group2	Statistic	Test	MeanRank1	MeanRank2	p_adjusted	significant	effect_size	effect_size_type
I7_2022	T1_P1→T2_P2	T1_P2→T2_P1	8.01	Dunn'sz	692.86	461.77	<.001	Yes	0.05	rank-eta²
I7_2022	T1_P1→T2_P2	T1_P2→T2_P2	-2.11	Dunn'sz	692.86	747.67	.211	No	0.00	rank-eta²
I7_2022	T1_P2→T2_P1	T1_P2→T2_P2	-11.11	Dunn'sz	461.77	747.67	<.001	Yes	0.10	rank-eta²
E5_2022	T1_P1→T2_P1	T1_P1→T2_P2	-8.14	Dunn'sz	459.93	682.71	<.001	Yes	0.06	rank-eta²
E5_2022	T1_P1→T2_P1	T1_P2→T2_P1	-0.92	Dunn'sz	459.93	484.91	1.000	No	0.00	rank-eta²
E5_2022	T1_P1→T2_P1	T1_P2→T2_P2	-11.36	Dunn'sz	459.93	733.04	<.001	Yes	0.11	rank-eta²
E5_2022	T1_P1→T2_P2	T1_P2→T2_P1	6.85	Dunn'sz	682.71	484.91	<.001	Yes	0.04	rank-eta²
E5_2022	T1_P1→T2_P2	T1_P2→T2_P2	-1.93	Dunn'sz	682.71	733.04	.320	No	0.00	rank-eta²
E5_2022	T1_P2→T2_P1	T1_P2→T2_P2	-9.64	Dunn'sz	484.91	733.04	<.001	Yes	0.08	rank-eta²
E6_2022	T1_P1→T2_P1	T1_P1→T2_P2	-4.48	Dunn'sz	517.43	627.83	<.001	Yes	0.02	rank-eta²
E6_2022	T1_P1→T2_P1	T1_P2→T2_P1	-1.78	Dunn'sz	517.43	560.75	.454	No	0.00	rank-eta²
E6_2022	T1_P1→T2_P1	T1_P2→T2_P2	-7.22	Dunn'sz	517.43	673.81	<.001	Yes	0.04	rank-eta²
E6_2022	T1_P1→T2_P2	T1_P2→T2_P1	2.58	Dunn'sz	627.83	560.75	.059	No	0.01	rank-eta²
E6_2022	T1_P1→T2_P2	T1_P2→T2_P2	-1.96	Dunn'sz	627.83	673.81	.300	No	0.00	rank-eta²
E6_2022	T1_P2→T2_P1	T1_P2→T2_P2	-4.87	Dunn'sz	560.75	673.81	<.001	Yes	0.02	rank-eta²
I8_2022	T1_P1→T2_P1	T1_P1→T2_P2	-4.17	Dunn'sz	541.63	655.67	<.001	Yes	0.01	rank-eta²
I8_2022	T1_P1→T2_P1	T1_P2→T2_P1	0.48	Dunn'sz	541.63	528.75	1.000	No	0.00	rank-eta²
I8_2022	T1_P1→T2_P1	T1_P2→T2_P2	-4.84	Dunn'sz	541.63	658.09	<.001	Yes	0.02	rank-eta²
I8_2022	T1_P1→T2_P2	T1_P2→T2_P1	4.39	Dunn'sz	655.67	528.75	<.001	Yes	0.02	rank-eta²
I8_2022	T1_P1→T2_P2	T1_P2→T2_P2	-0.09	Dunn'sz	655.67	658.09	1.000	No	0.00	rank-eta²
I8_2022	T1_P2→T2_P1	T1_P2→T2_P2	-5.02	Dunn'sz	528.75	658.09	<.001	Yes	0.02	rank-eta²

Note. Kruskal-Wallis *H* tests comparing item scores across four transition groups (stable Lower Profile, Lower-to-Higher, Higher-to-Lower, stable Higher Profile) at both Time 1 and Time 2. Post-hoc pairwise comparisons following a significant Kruskal-Wallis *H* test.

Table S4. Betweennes Centrality Indices

Items	*Time 1*				*Time 2*			
	Lower-severity		Higher-severity		Lower-severity		Higher-severity	
	Betweennes	95% CI	Betweennes	95% CI	Betweennes	95% CI	Betweennes	95% CI
I1	0.00	/	0.00	[0.00, 30.00]	0.00	[0.00, 22.02]	27.00	[0.00, 35.00]
E1	1.00	[0.00, 27.02]	32.00	[0.00, 44.00]	4.00	[0.00, 29.02]	20.00	[0.00, 36.02]
I2	0.00	[0.00, 9.00]	36.00	[0.00, 46.00]	14.00	[0.00, 31.00]	40.00	[0.00, 48.00]
E2	0.00	[0.00, 20.00]	40.00	[0.00, 34.02]	0.00	[0.00, 22.00]	13.00	[0.00, 25.02]
I3	8.00	[0.00, 26.02]	0.00	[0.00, 19.00]	26.00	[0.00, 32.00]	5.00	[0.00, 33.00]
E3	0.00	[0.00, 27.00]	42.00	[0.00, 42.00]	0.00	[0.00, 19.00]	6.00	[0.00, 32.00]
I4	4.00	[0.00, 17.02]	12.00	[0.00, 44.00]	6.00	[0.00, 23.00]	2.00	[0.00, 34.00]
E4	11.00	[0.00, 39.00]	50.00	[0.00, 50.00]	28.00	[1.00, 37.02]	0.00	[0.00, 3.05]
I5	8.00	[0.00, 30.05]	12.00	[0.00, 42.00]	24.00	[0.00, 33.00]	23.00	[0.00, 40.00]
I6	14.00	[0.00, 39.00]	32.00	[0.00, 47.00]	0.00	[0.00, 17.00]	0.00	[0.00, 32.02]
I7	19.00	[0.00, 41.00]	0.00	[0.00, 22.00]	0.00	[0.00, 11.00]	14.00	[0.00, 46.00]
E5	0.00	[0.00, 16.00]	0.00	[0.00, 35.00]	1.00	[0.00, 30.00]	0.00	[0.00, 25.00]
E6	0.00	[0.00, 20.02]	0.00	[0.00, 25.05]	0.00	[0.00, 17.00]	11.00	[0.00, 25.00]
I8	0.00	[0.00, 20.00]	0.00	[0.00, 36.00]	0.00	[0.00, 12.00]	0.00	[0.00, 22.00]

Note. Betweenness centrality indices with 95% bootstrap CIs for all 14 nodes across 4 profiles at Time 1 and Time 2. Based on 1,000 bootstrap samples. Betweenness and closeness values are included here for completeness but are unstable (CS < 0.50). "/" indicates failure in estimating 95% CI due to zero variance.

Table S5. Closeness Centrality Indices

Items	*Time 1*				*Time 2*			
	Lower-severity		Higher-severity		Lower-severity		Higher-severity	
	Closeness	95% CI	Closeness	95% CI	Closeness	95% CI	Closeness	95% CI
I1	0.00	[0.00, 13.43]	2.33	[0.00, 6.53]	3.51	[0.00, 7.57]	3.33	[2.29, 6.10]
E1	4.01	[2.30, 7.64]	2.68	[1.80, 8.74]	5.12	[3.82, 8.42]	2.76	[2.09, 6.26]
I2	0.00	[0.00, 14.48]	2.86	[1.89, 7.16]	4.88	[3.45, 7.91]	4.03	[2.70, 7.08]
E2	3.30	[1.37, 5.56]	3.00	[1.96, 5.99]	4.41	[1.91, 6.90]	3.54	[1.29, 5.20]
I3	4.74	[2.57, 6.93]	1.92	[1.77, 6.16]	6.95	[3.58, 8.46]	2.67	[1.98, 5.61]
E3	3.30	[1.75, 5.97]	3.33	[2.13, 6.80]	5.18	[1.65, 7.05]	2.19	[1.77, 6.13]
I4	4.61	[2.78, 7.26]	2.78	[2.16, 6.99]	4.98	[3.88, 7.16]	2.07	[1.76, 6.41]
E4	5.40	[2.90, 8.51]	3.33	[2.57, 6.92]	6.83	[4.27, 8.53]	0.00	[0.00, 4.28]
I5	3.17	[2.06, 6.02]	1.97	[2.14, 6.29]	6.06	[3.32, 7.90]	3.56	[2.42, 6.54]
I6	4.37	[2.99, 7.18]	2.77	[2.84, 6.49]	4.66	[3.07, 6.93]	3.40	[2.35, 5.93]
I7	5.66	[3.57, 7.69]	2.26	[0.00, 5.38]	4.35	[3.68, 6.69]	3.30	[2.29, 7.28]
E5	4.66	[2.63, 6.84]	2.31	[1.62, 7.25]	6.09	[3.82, 8.28]	1.75	[0.00, 6.36]
E6	4.26	[2.69, 6.75]	1.92	[1.87, 5.92]	5.21	[3.81, 7.31]	2.16	[1.86, 6.78]
I8	2.72	[0.00, 5.64]	1.61	[1.49, 5.81]	4.21	[0.00, 6.14]	2.65	[0.00, 5.66]

Note. Closeness centrality indices with 95% bootstrap CIs for all 14 nodes across 4 profiles at Time 1 and Time 2. Based on 1,000 bootstrap samples. Betweenness and closeness values are included here for completeness but are unstable (CS < 0.50).

Table S6. Network Comparison Test Omnibus Results

Wave	n lower	n higher	Structure *M*	Structure *p*	Global strength lower	Global strength higher	Global strength *S*	Global strength *p*
Time 1	552	648	0.219	0.014	2.596	0.850	1.747	0.004
Time 2	561	639	0.183	0.100	4.258	1.846	2.412	< .001

Note. Structure M is the maximum absolute difference between corresponding edge weights across the two networks. Global strength is the sum of absolute edge weights. Global strength *S* is the absolute difference in global strength between the two profiles. Lower and higher refer to the lower-symptom and higher-symptom profiles. All *p* values are permutation-based.

Table S7. Network Comparison Test Edge-Level Results

Wave	Edge	Weight lower	Weight higher	Difference	*p* (raw)	*p* (Holm)
Time 1	I1-E1	0.018	0.000	0.018	0.777	1.000
Time 1	I1-I2	0.000	0.000	0.000	1.000	1.000
Time 1	E1-I2	0.000	0.000	0.000	1.000	1.000
Time 1	I1-E2	0.000	0.000	0.000	1.000	1.000
Time 1	E1-E2	0.000	0.000	0.000	1.000	1.000
Time 1	I2-E2	0.000	0.000	0.000	1.000	1.000
Time 1	I1-I3	0.000	0.000	0.000	1.000	1.000
Time 1	E1-I3	0.061	0.000	0.061	0.324	1.000
Time 1	I2-I3	0.000	0.000	0.000	1.000	1.000
Time 1	E2-I3	0.000	0.000	0.000	1.000	1.000
Time 1	I1-E3	0.000	0.000	0.000	1.000	1.000
Time 1	E1-E3	0.000	0.000	0.000	1.000	1.000
Time 1	I2-E3	0.000	0.000	0.000	1.000	1.000
Time 1	E2-E3	0.310	0.156	0.154	0.012	1.000
Time 1	I3-E3	0.000	0.000	0.000	1.000	1.000
Time 1	I1-I4	0.000	0.000	0.000	1.000	1.000
Time 1	E1-I4	0.000	0.000	0.000	1.000	1.000
Time 1	I2-I4	0.000	0.000	0.000	1.000	1.000
Time 1	E2-I4	0.000	0.000	0.000	1.000	1.000
Time 1	I3-I4	0.135	0.106	0.029	0.674	1.000
Time 1	E3-I4	0.000	0.000	0.000	1.000	1.000
Time 1	I1-E4	0.000	0.000	0.000	1.000	1.000
Time 1	E1-E4	0.031	0.000	0.031	0.590	1.000
Time 1	I2-E4	0.000	0.000	0.000	1.000	1.000
Time 1	E2-E4	0.028	0.000	0.028	0.669	1.000
Time 1	I3-E4	0.064	0.000	0.064	0.331	1.000
Time 1	E3-E4	0.000	0.008	0.008	0.895	1.000
Time 1	I4-E4	0.032	0.013	0.019	0.789	1.000
Time 1	I1-I5	0.000	0.000	0.000	1.000	1.000
Time 1	E1-I5	0.000	0.000	0.000	1.000	1.000
Time 1	I2-I5	0.000	0.000	0.000	1.000	1.000

Time 1	E2-I5	0.000	0.000	0.000	1.000	1.000
Time 1	I3-I5	0.000	0.000	0.000	1.000	1.000
Time 1	E3-I5	0.029	0.000	0.029	0.634	1.000
Time 1	I4-I5	0.000	0.000	0.000	1.000	1.000
Time 1	E4-I5	0.000	0.000	0.000	1.000	1.000
Time 1	I1-I6	0.000	0.000	0.000	1.000	1.000
Time 1	E1-I6	0.000	0.000	0.000	1.000	1.000
Time 1	I2-I6	0.000	0.000	0.000	1.000	1.000
Time 1	E2-I6	0.000	0.000	0.000	1.000	1.000
Time 1	I3-I6	0.000	0.000	0.000	1.000	1.000
Time 1	E3-I6	0.005	0.000	0.005	0.928	1.000
Time 1	I4-I6	0.000	0.000	0.000	1.000	1.000
Time 1	E4-I6	0.042	0.072	0.030	0.641	1.000
Time 1	I5-I6	0.137	0.128	0.009	0.875	1.000
Time 1	I1-I7	0.000	0.000	0.000	1.000	1.000
Time 1	E1-I7	0.027	0.000	0.027	0.198	1.000
Time 1	I2-I7	0.000	0.000	0.000	1.000	1.000
Time 1	E2-I7	0.000	0.000	0.000	1.000	1.000
Time 1	I3-I7	0.187	0.000	0.187	< .001	0.090
Time 1	E3-I7	0.000	0.000	0.000	1.000	1.000
Time 1	I4-I7	0.119	0.000	0.119	0.080	1.000
Time 1	E4-I7	0.107	0.000	0.107	0.046	1.000
Time 1	I5-I7	0.000	0.000	0.000	1.000	1.000
Time 1	I6-I7	0.122	0.022	0.100	0.114	1.000
Time 1	I1-E5	0.000	0.000	0.000	1.000	1.000
Time 1	E1-E5	0.036	0.020	0.015	0.824	1.000
Time 1	I2-E5	0.000	0.000	0.000	1.000	1.000
Time 1	E2-E5	0.000	0.000	0.000	1.000	1.000
Time 1	I3-E5	0.000	0.000	0.000	1.000	1.000
Time 1	E3-E5	0.000	0.000	0.000	1.000	1.000
Time 1	I4-E5	0.000	0.000	0.000	1.000	1.000
Time 1	E4-E5	0.057	0.000	0.057	0.363	1.000
Time 1	I5-E5	0.000	0.000	0.000	1.000	1.000
Time 1	I6-E5	0.000	0.000	0.000	1.000	1.000
Time 1	I7-E5	0.123	0.000	0.123	0.057	1.000

Time 1	I1-E6	0.000	0.000	0.000	1.000	1.000
Time 1	E1-E6	0.132	0.109	0.024	0.750	1.000
Time 1	I2-E6	0.000	0.000	0.000	1.000	1.000
Time 1	E2-E6	0.000	0.000	0.000	1.000	1.000
Time 1	I3-E6	0.038	0.000	0.038	0.284	1.000
Time 1	E3-E6	0.000	0.000	0.000	1.000	1.000
Time 1	I4-E6	0.067	0.000	0.067	0.016	1.000
Time 1	E4-E6	0.142	0.000	0.142	0.026	1.000
Time 1	I5-E6	0.000	0.000	0.000	1.000	1.000
Time 1	I6-E6	0.000	0.000	0.000	1.000	1.000
Time 1	I7-E6	0.219	0.000	0.219	< .001	0.018
Time 1	E5-E6	0.221	0.162	0.060	0.402	1.000
Time 1	I1-I8	0.000	0.000	0.000	1.000	1.000
Time 1	E1-I8	0.000	0.000	0.000	1.000	1.000
Time 1	I2-I8	0.000	0.000	0.000	1.000	1.000
Time 1	E2-I8	0.000	0.000	0.000	1.000	1.000
Time 1	I3-I8	0.000	0.000	0.000	1.000	1.000
Time 1	E3-I8	0.044	0.000	0.044	0.473	1.000
Time 1	I4-I8	0.000	0.000	0.000	1.000	1.000
Time 1	E4-I8	0.000	0.000	0.000	1.000	1.000
Time 1	I5-I8	0.064	0.056	0.009	0.883	1.000
Time 1	I6-I8	0.000	0.000	0.000	1.000	1.000
Time 1	I7-I8	0.000	0.000	0.000	1.000	1.000
Time 1	E5-I8	0.000	0.000	0.000	1.000	1.000
Time 1	E6-I8	0.000	0.000	0.000	1.000	1.000
Time 2	I1-E1	0.040	0.087	0.046	0.409	1.000
Time 2	I1-I2	0.088	0.110	0.022	0.704	1.000
Time 2	E1-I2	0.017	0.000	0.017	0.778	1.000
Time 2	I1-E2	0.036	0.000	0.036	0.550	1.000
Time 2	E1-E2	0.000	0.000	0.000	1.000	1.000
Time 2	I2-E2	0.011	0.040	0.029	0.636	1.000
Time 2	I1-I3	0.018	0.000	0.018	0.499	1.000
Time 2	E1-I3	0.106	0.019	0.086	0.217	1.000
Time 2	I2-I3	0.000	0.000	0.000	1.000	1.000
Time 2	E2-I3	0.031	0.000	0.031	0.577	1.000

Time 2	I1-E3	0.008	0.000	0.008	0.888	1.000
Time 2	E1-E3	0.000	0.000	0.000	1.000	1.000
Time 2	I2-E3	0.036	0.000	0.036	0.553	1.000
Time 2	E2-E3	0.339	0.254	0.085	0.138	1.000
Time 2	I3-E3	0.000	0.011	0.011	0.837	1.000
Time 2	I1-I4	0.000	0.000	0.000	1.000	1.000
Time 2	E1-I4	0.127	0.000	0.127	0.079	1.000
Time 2	I2-I4	0.114	0.000	0.114	0.037	1.000
Time 2	E2-I4	0.000	0.000	0.000	1.000	1.000
Time 2	I3-I4	0.227	0.225	0.002	0.979	1.000
Time 2	E3-I4	0.000	0.076	0.076	0.154	1.000
Time 2	I1-E4	0.000	0.000	0.000	1.000	1.000
Time 2	E1-E4	0.046	0.000	0.046	0.507	1.000
Time 2	I2-E4	0.000	0.000	0.000	1.000	1.000
Time 2	E2-E4	0.088	0.000	0.088	0.140	1.000
Time 2	I3-E4	0.065	0.000	0.065	0.245	1.000
Time 2	E3-E4	0.051	0.000	0.051	0.351	1.000
Time 2	I4-E4	0.183	0.000	0.183	< .001	0.036
Time 2	I1-I5	0.021	0.021	0.001	0.991	1.000
Time 2	E1-I5	0.000	0.000	0.000	1.000	1.000
Time 2	I2-I5	0.093	0.109	0.016	0.790	1.000
Time 2	E2-I5	0.000	0.000	0.000	1.000	1.000
Time 2	I3-I5	0.070	0.000	0.070	0.198	1.000
Time 2	E3-I5	0.046	0.000	0.046	0.394	1.000
Time 2	I4-I5	0.000	0.000	0.000	1.000	1.000
Time 2	E4-I5	0.000	0.000	0.000	1.000	1.000
Time 2	I1-I6	0.000	0.000	0.000	1.000	1.000
Time 2	E1-I6	0.000	0.000	0.000	1.000	1.000
Time 2	I2-I6	0.000	0.104	0.104	0.076	1.000
Time 2	E2-I6	0.015	0.000	0.015	0.794	1.000
Time 2	I3-I6	0.024	0.000	0.024	0.719	1.000
Time 2	E3-I6	0.000	0.000	0.000	1.000	1.000
Time 2	I4-I6	0.000	0.000	0.000	1.000	1.000
Time 2	E4-I6	0.093	0.000	0.093	0.139	1.000
Time 2	I5-I6	0.176	0.212	0.036	0.551	1.000

Time 2	I1-I7	0.000	0.000	0.000	1.000	1.000
Time 2	E1-I7	0.036	0.000	0.036	0.543	1.000
Time 2	I2-I7	0.015	0.005	0.010	0.870	1.000
Time 2	E2-I7	0.000	0.000	0.000	1.000	1.000
Time 2	I3-I7	0.195	0.106	0.089	0.200	1.000
Time 2	E3-I7	0.000	0.000	0.000	1.000	1.000
Time 2	I4-I7	0.187	0.025	0.162	0.020	1.000
Time 2	E4-I7	0.135	0.000	0.135	0.003	0.234
Time 2	I5-I7	0.000	0.054	0.054	0.367	1.000
Time 2	I6-I7	0.214	0.064	0.150	0.009	0.757
Time 2	I1-E5	0.000	0.000	0.000	1.000	1.000
Time 2	E1-E5	0.088	0.000	0.088	0.041	1.000
Time 2	I2-E5	0.043	0.000	0.043	0.187	1.000
Time 2	E2-E5	0.000	0.000	0.000	1.000	1.000
Time 2	I3-E5	0.050	0.000	0.050	0.098	1.000
Time 2	E3-E5	0.000	0.000	0.000	1.000	1.000
Time 2	I4-E5	0.047	0.000	0.047	0.484	1.000
Time 2	E4-E5	0.105	0.000	0.105	0.020	1.000
Time 2	I5-E5	0.031	0.000	0.031	0.628	1.000
Time 2	I6-E5	0.000	0.000	0.000	1.000	1.000
Time 2	I7-E5	0.152	0.025	0.127	0.044	1.000
Time 2	I1-E6	0.000	0.000	0.000	1.000	1.000
Time 2	E1-E6	0.073	0.112	0.039	0.640	1.000
Time 2	I2-E6	0.006	0.000	0.006	0.924	1.000
Time 2	E2-E6	0.000	0.000	0.000	1.000	1.000
Time 2	I3-E6	0.040	0.000	0.040	0.423	1.000
Time 2	E3-E6	0.000	0.000	0.000	1.000	1.000
Time 2	I4-E6	0.094	0.000	0.094	0.147	1.000
Time 2	E4-E6	0.131	0.000	0.131	0.030	1.000
Time 2	I5-E6	0.000	0.000	0.000	1.000	1.000
Time 2	I6-E6	0.000	0.000	0.000	1.000	1.000
Time 2	I7-E6	0.169	0.000	0.169	0.005	0.409
Time 2	E5-E6	0.153	0.100	0.052	0.458	1.000
Time 2	I1-I8	0.036	0.000	0.036	0.544	1.000
Time 2	E1-I8	-0.007	0.000	0.007	0.657	1.000

Time 2	I2-I8	0.000	0.000	0.000	1.000	1.000
Time 2	E2-I8	0.000	0.000	0.000	1.000	1.000
Time 2	I3-I8	0.000	0.000	0.000	1.000	1.000
Time 2	E3-I8	0.000	0.000	0.000	1.000	1.000
Time 2	I4-I8	0.000	0.000	0.000	1.000	1.000
Time 2	E4-I8	0.000	0.000	0.000	1.000	1.000
Time 2	I5-I8	0.081	0.086	0.005	0.927	1.000
Time 2	I6-I8	0.000	0.000	0.000	1.000	1.000
Time 2	I7-I8	0.000	0.000	0.000	1.000	1.000
Time 2	E5-I8	0.000	0.000	0.000	1.000	1.000
Time 2	E6-I8	0.000	0.000	0.000	1.000	1.000

Note. Difference is the absolute difference in edge weight between the lower-symptom and higher-symptom networks. The Holm correction was applied across the 91 edge tests separately within each wave. I1 through I8 denote internalizing items and E1 through E6 denote externalizing items.

Table S8. Network Comparison Test Node-Strength Results

Wave	Node	Strength lower	Strength higher	Difference	p (raw)	p (Holm)
Time 1	I1	0.018	0.000	0.018	0.899	1.000
Time 1	E1	0.305	0.129	0.176	0.184	1.000
Time 1	I2	0.000	0.000	0.000	1.000	1.000
Time 1	E2	0.338	0.156	0.182	0.130	0.911
Time 1	I3	0.485	0.106	0.380	0.010	0.108
Time 1	E3	0.388	0.164	0.224	0.090	0.721
Time 1	I4	0.352	0.118	0.234	0.070	0.632
Time 1	E4	0.503	0.093	0.411	0.005	0.060
Time 1	I5	0.230	0.183	0.047	0.733	1.000
Time 1	I6	0.305	0.221	0.084	0.501	1.000
Time 1	I7	0.904	0.022	0.882	< .001	0.003
Time 1	E5	0.437	0.182	0.255	0.059	0.592
Time 1	E6	0.819	0.270	0.549	< .001	0.003
Time 1	I8	0.109	0.056	0.053	0.775	1.000
Time 2	I1	0.247	0.218	0.030	0.789	1.000
Time 2	E1	0.541	0.218	0.323	0.021	0.171
Time 2	I2	0.423	0.368	0.055	0.594	1.000
Time 2	E2	0.519	0.294	0.225	0.037	0.262
Time 2	I3	0.826	0.361	0.464	< .001	0.003
Time 2	E3	0.480	0.341	0.139	0.215	1.000
Time 2	I4	0.980	0.325	0.654	< .001	0.003
Time 2	E4	0.898	0.000	0.898	< .001	0.003
Time 2	I5	0.518	0.482	0.035	0.743	1.000
Time 2	I6	0.521	0.381	0.141	0.227	1.000
Time 2	I7	1.103	0.279	0.824	< .001	0.003
Time 2	E5	0.670	0.126	0.545	< .001	0.003
Time 2	E6	0.666	0.212	0.454	< .001	0.007
Time 2	I8	0.124	0.086	0.038	0.800	1.000

Note. Strength is the sum of the absolute edge weights connected to a node. Difference is the absolute difference in node strength between the lower-symptom and higher-symptom networks. The Holm correction was applied across the 14 node tests separately within each wave.

Table S9. Network Summary by Gamma Value

Wave	Profile	Gamma	Retained edges	Density	Global strength	Top 1	Top 2	Top 3
Time 1	Higher	0.00	18	0.198	2.018	I6	E6	E3
Time 1	Higher	0.25	14	0.154	1.669	I6	E6	E1
Time 1	Higher	0.50	14	0.154	1.669	I6	E6	E1
Time 1	Lower	0.00	25	0.275	3.193	I7	E6	E4
Time 1	Lower	0.25	23	0.253	2.905	I7	E6	E4
Time 1	Lower	0.50	17	0.187	2.214	I7	E6	I3
Time 2	Higher	0.00	24	0.264	3.068	I5	I3	E3
Time 2	Higher	0.25	18	0.198	2.436	I5	I2	I6
Time 2	Higher	0.50	15	0.165	1.986	I5	I6	I2
Time 2	Lower	0.00	31	0.341	4.003	I7	I4	E4
Time 2	Lower	0.25	28	0.308	3.630	I7	I4	E4
Time 2	Lower	0.50	28	0.308	3.630	I7	I4	E4

Note. Retained edges were counted at an absolute edge-weight threshold of .05. Density is the proportion of the 91 possible edges that were retained. Global strength is the sum of the absolute edge weights. Top 1 through Top 3 are the three nodes with the highest strength centrality, in descending order. The gamma = .50 column reproduces the primary analysis reported in the main text.

Figure S1. CS Coefficients for Centrality Indices of Time 1

Lower-severity

Higher-severity

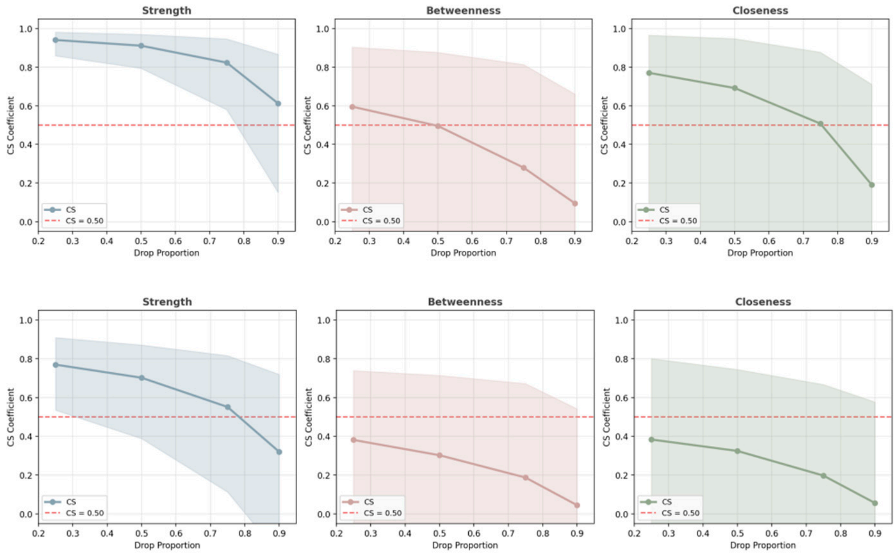

Note. Obtained through 1000 bootstrap sampling. The horizontal axis represents sampled cases. The vertical axis in the left figure shows the CS coefficients of centrality indices. Average CS for strength, betweenness and closeness were .94, .60 and .77 respectively for lower-severity profile; and were .77, .38 and .38 respectively for higher-severity profile.

Figure S2. CS Coefficients for Centrality Indices of Time 2

Lower-severity

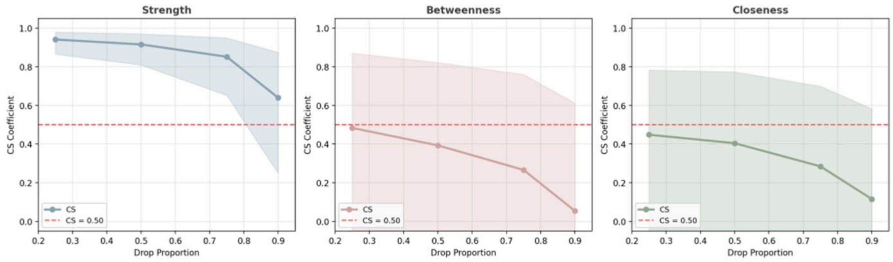

Higher-severity

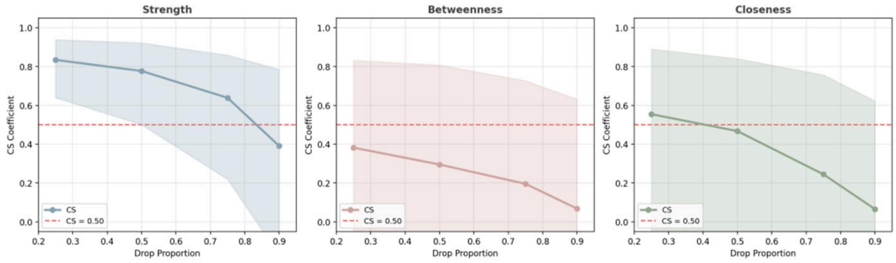

Note. Obtained through 1000 bootstrap sampling. The horizontal axis represents sampled cases. The vertical axis in the left figure shows the CS coefficients of centrality indices. Average CS for strength, betweenness and closeness were .94, .48 and .45 respectively for lower-severity profile; and were .83, .38 and .55 respectively for higher-severity profile.

Figure S3. The estimated profile solutions for the two-class through five-class models.

a.

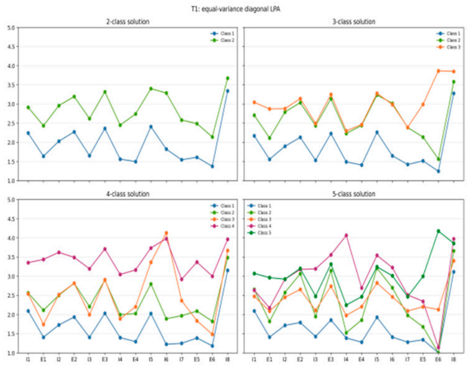

b.

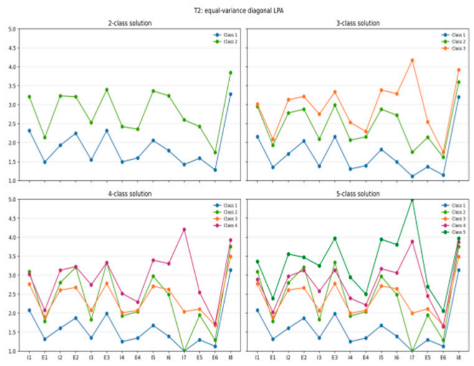

Note. Estimated item means are plotted for the retained two-class solution and the three-class, four-class, and five-class alternatives at each wave. I1 through I8 denote internalizing items and E1 through E6 denote externalizing items.

Figure S4. Network Comparison Test Permutation Distributions

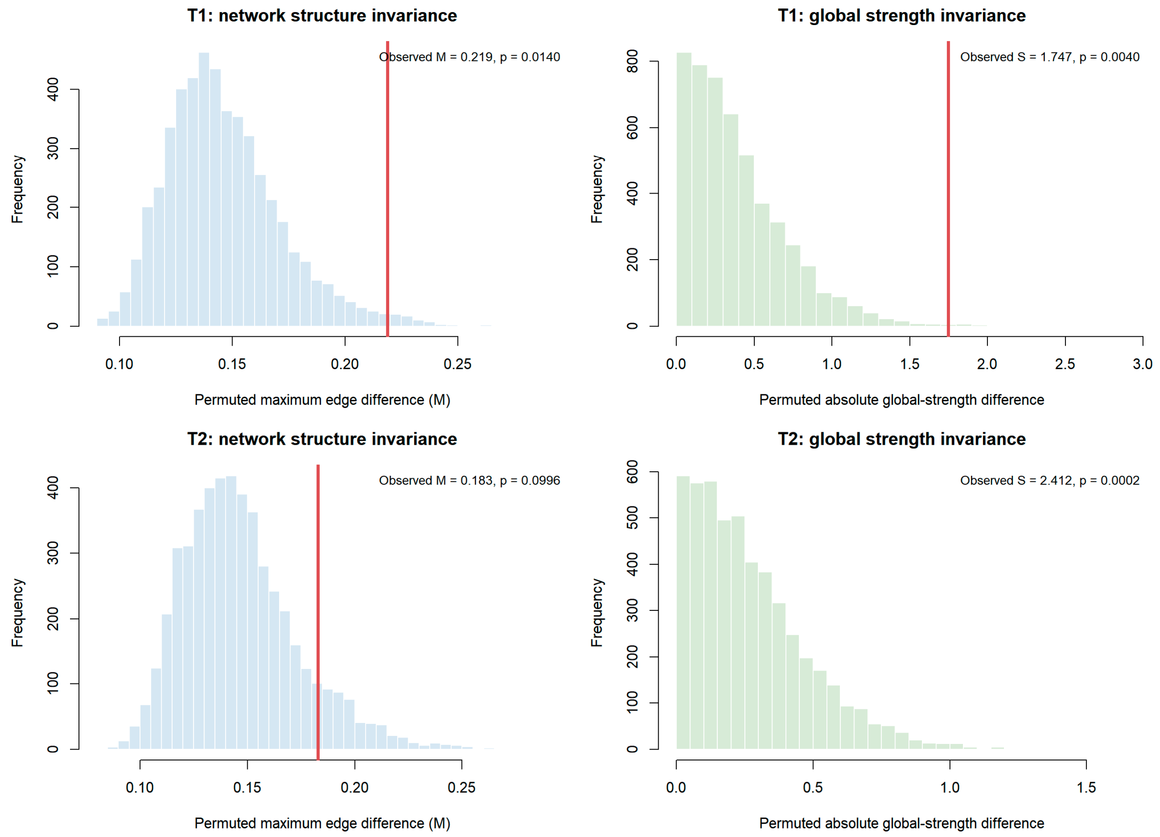

Note. Permutation distributions of the network-structure statistic M and the global-strength difference S for the Time 1 and Time 2 comparisons. Each distribution is based on 5,000 independent-sample label permutations. The vertical lines mark the observed statistics; the reported *p* values are the proportions of permuted statistics that were at least as extreme as the observed value.
